# BIRC5 regulates inflammatory tumor microenvironment-induced aggravation of penile cancer development *in vitro* and *in vivo*

**DOI:** 10.1186/s12885-022-09500-9

**Published:** 2022-04-23

**Authors:** Yang Zhao, Songlin Liu, Shuhang Li, Gang Zhang, Aimin Tian, Yinxu Wan

**Affiliations:** Department of Urology, Yantai Affiliated Hospital of Binzhou Medical University, No. 717 Jinbu Street, Muping DistinctYantai, 264100 Shandong China

**Keywords:** BIRC5, Inflammation, Tumor microenvironment, Penile cancer, Migration and invasion

## Abstract

**Background:**

Baculoviral IAP repeat containing 5 (BIRC5) is overexpressed and plays as a key regulator in the progression of various human carcinomas. The inflammatory tumor microenvironment (ITM) is closely associated with the development of cancers. However, the role of BIRC5 in penile cancer (PC) and the ITM-induced abnormal progression of PC is still obscure.

**Methods:**

In this study, serum and tissues of patients with PC were recruited to evaluate the expression profile of BIRC5. We used PC cell lines (Penl1 and Penl2) and constructed a PC xenograft mice model to explore the effects of the silencing of BIRC5 on proliferation, migration, invasion and tumor growth, as well as survival of mice. Besides, interferon (IFN)-γ was utilized to mimic the ITM of PC cells.

**Results:**

Our results showed that BIRC5 was dramatically upregulated in the serum and tissues of PC patients, as well as PC cell lines. Knockdown of BIRC5 inhibited the proliferation, migration and invasion of PC cells. Meanwhile, it suppressed PC xenograft tumor growth and improved mice survival. Moreover, IFN-γ significantly aggravated PC progression both *in vivo* and *in vitro* while the silencing of BIRC5 reversed these unfavorable effects.

**Conclusions:**

Taken together, our data revealed that BIRC5 silencing inhibited aggravation of PC cell processes and tumor development induced by ITM. This suggested that BIRC5 may function as a diagnosis and therapy target of PC in the future.

**Supplementary Information:**

The online version contains supplementary material available at 10.1186/s12885-022-09500-9.

## Background

Penile cancer (PC) is an easily overlooked and aggressive cancer in economically undeveloped countries [1, 2]. In all, 25% of PC patients are initially diagnosed as a late stage cancer due to insufficient emphasis and embarrassment [3]. Currently, clinical treatment approaches for PC include surgery, chemotherapy and brachytherapy [2, 4, 5]. Although clinical treatments effectively slow the progression of disease, the survival of PC patients is still low [6–8]. Patients with pelvic nodal metastasis even have a 0% 5-year overall survival rate [9, 10]. A better understanding of biomarkers related to PC is urgently needed for cancer treatment.

Baculoviral IAP repeat containing 5 (BIRC5), also referred to as survivin, was first reported in 1997 and discovered as a member of the inhibitor of apoptosis proteins (IAPs) family which is located on the 17q25 chromosome of humans [11, 12]. So far, there were numerous evidence indicated that hyperactivation of BIRC5 was occurred in various tumor diseases and played an oncogenic role in carcinogenesis [13]. Conde et al*.* demonstrated that BIRC5 affected cancer aggressiveness by depressing apoptosis-related pathways, which led to the promotion of cell proliferation [14]. The increased expression of BIRC5 was associated with markers of tumor histological malignancy and poor patient prognosis in gliomas [15]. A recent study based on TCGA dataset and hospital data showed that BIRC5 was highly expressed in breast cancer tissues compared with normal individuals and may be adopted as a promising therapeutic bio-target [16]. However, there is a lack of study which exactly illustrate the role and function of BIRC5 during PC pathogenesis.

Tumor microenvironment refers to the occurrence, growth and metastasis of tumors and the internal and external environment in which tumor cells are located [17]. Immune cell infiltration has been demonstrated to exist in the tumor microenvironment and the inflammatory cytokines secreted by them play a key role in regulating the tumor growth and development of multiple tumor diseases, including PC [18, 19]. Recently, some types of pro-inflammatory factors have been adopted to predict the outcome of patients [20]. Anuja and colleagues indicated that persistent exposure to inflammation was closely connected to the pro-neoplasm of PC tumor [21]. Moreover, large amounts of inflammatory penile diseases are regarded to have a high probability of eventually developing into PC [22]. Therefore, exploring the potential regulatory mechanism of inflammatory tumor microenvironment (ITM) on PC progression is very necessary.

In our research, PC cell lines and a PC xenograft tumor mice model were utilized to investigated the expression and function of BIRC5 in PC development. Moreover, we further explored the involvement of BIRC5 in ITM-induced PC aggravation. Our findings are expected to provide a novel approach for PC diagnosis and therapy.

## Methods

### Patient sample collection and ethic approval

Our study enrolled 27 cases of serum samples from PC patients (age: 32 ~ 69 years old, average: 52 years old) and equal amounts of serum from healthy subjects (age: 29 ~ 72 years old, average: 50 years old). PC patients were pathologically diagnosed as penile squamous cell carcinoma with 15 cases of inguinal lymph node metastasis. The clinical staging of TMN was conducted according to the WHO pathological stage method. All the patients, who had undergone brachytherapy or chemotherapy before, were eliminated were diagnosed at Yantai Affiliated Hospital of Binzhou Medical University from March 2014 to October 2018 and clinically managed in line with NCCN guideline of PS. The specific therapy that PC patients received was consistent with a previous study [23]. The whole study conformed to the Declaration of Helsinki. Besides, seven cases of patients (age: 48 ~ 69 years old, average: 55 years old) were received penectomy and collected samples of PC and adjacent tissues. The patients featured as lymph node metastasis and the tissues were collected from a lymph node while the adjacent tissues were matched, 2 cm away from tumor sites. The tissues were immediately frozen and stored at -80℃ after surgery. All the patients provided written informed consents and our study obtained approval from the Institutional Research Ethic Committee of Yantai Affiliated Hospital of Binzhou Medical University.

### Cell culture and inflammatory treatment

Human epidermis keratinocyte cells (HaCaT) were used as the normal control and obtained from the National Infrastructure of Cell Line Resource (Wuhan, China). PC cell lines (Penl1 and Penl2) were kindly provided from Department of Urology, Sun Yat-sen University Cancer Center. Cells were cultured in Dulbecco’s modified Eagle’s medium (DMEM) supplemented with 10% fetal bovine serum under a humidified atmosphere with 5% CO_2_ at 37 °C.

Interferon (IFN)-γ was purchased from Roche (Basel, Switzerland) and prepared at different concentrations. For concentration and time gradient screening experiments, IFN-γ was added to the medium after the cells were inoculated to a 96-well plate. To construct the inflammatory microenvironment, we used IFN-γ to treat PC cells which had been transfected with short hairpin RNA (shRNA) for an appropriate time and concentration. The transfection of shRNA was antecedent to IFN-γ treatment. And the transfection efficiency of shRNA was confirmed.

### Cell transfection

To silence BIRC5 expression in PC cells, shRNAs were designed and constructed by Invitrogen (CA, USA), and were connected into lentiviral vectors. Then, PC cells were seeded into 6-well plates at a concentration of 1 × 10^5^ cells/well and cultured to 2 × 10^5^ cells/well. The next day, the medium was replaced by fresh medium which supplemented with 6 μg/mL polybrene. Then, the lentiviral suspension was added and incubated PC cells at 37 °C for 72 h. Subsequently, the cells were screened 10 U/mL using ampicillin. After the incubation, the transfected samples were collected, and the effectiveness of transfection and subsequent function detections were evaluated.

### Xenograft mice construction

After being approved by the Animal Ethical Committee of Yantai Affiliated Hospital of Binzhou Medical University, BALB/c nude mice (6 weeks old, 17 g ~ 23 g; provided by Vital River, Bejing, China) were raised to adapt to the experimental environment for about 7 days. They were free to access chow and water in a cage with no pathogens. To construct the xenograft model, HaCaT cells (control), Penl1 cells under different treatments (scrambled shRNA; BIRC5 shRNAb, IFN-γ; IFN-γ + scrambled shRNA, IFN-γ + BIRC5 shRNAb) and Penl1 with any kind of treatment (model) were subcutaneously inoculated (100 µL containing 1 × 10^6^ cells) at the right axilla after the mice were anesthetized. Tumor size and weight were measured throughout the tumor growth process and tumor volume was calculated. Besides, the survival of mice was recorded. The whole research was in accordance with the Health Guide for the Care and Use of Laboratory Animals (National Institutes) and adhered to the ARRIVE guidelines.

### Western blot assay

The protein expressions in PC cells were all evaluated by standard procedures as described previously [24, 25]. The information of antibodies was as follows: rabbit polyclonal to BIRC antibody (ab76424; 1: 1000), rabbit monoclonal to matrix metalloproteinase 2 (MMP2) antibody (ab92536; 1: 1000), rabbit monoclonal to MMP9 antibody (ab76003; 1: 1000), rabbit monoclonal to E-cadherin antibody (ab40772; 1:1000) and rabbit monoclonal to β-actin antibody (ab8227; 1:2000). All antibodies were obtained from Abcam (Cambridge, MA, USA). The blotting signals were visualized by chemiluminescence reagents (Millipore, MA, USA). The quantification of protein bands was performed using Image J software.

### RT-qPCR

For profiling the mRNA expressions of PC cells, a TRIzol kit purchased from Invitrogen (CA, USA) was used to extract total RNAs. After purification and quantification, 50 ng RNA was reverse-transcribed into a first-stand cDNA in line with the protocol of the High-Capacity cDNA Reverse Transcription Kit (Applied Biosystems, Foster City, USA). Then, on an ABI Sequence Detection System (7500, Applied Biosystems, Foster City, USA), qPCR was performed. To calculated the final expression levels (relative) of target genes, we performed the 2^−ΔΔCt^ method and used β-actin as the reference gene. A plasmid containing the sequence of BIRC5 was set as a positive control to monitor whether the reaction system of RT-qPCR was normal.

### Cell viability detection

Cell viability was evaluated using the CCK-8 Kits (Beyotime, Shanghai, China) as previously described [26]. PC cells were seeded into 6‑well plates and treated with shRNAs and IFN-γ. After that, the CCK‑8 solution was added for another 1 h. The optical density (OD) value was measured at 450 nm by an auto microplate reader (Molecular Devices, USA).

### Migration and invasion

To evaluate the migration ability of PC cells, we seeded them at a density of 5 × 10^5^ cells per well in 6-well plates and incubated them for 48 h to reach the confluency. Then, a scratch wound was placed in the central well under sterile conditions. The slide wound distance was detected under a confocal microscopy (Roche, Basel, Switzerland), 24-h later.

The Transwell assay was performed to measure cell invasion. A Transwell chamber (8 µM, Sigma, St. Louis, USA) was pre-treated with Matrigel (50 µL) and PC cells were grown for 36 h at 37℃. Then, we fixed and stained the invaded cells on membrane using dehydrated alcohol and crystal violet, respectively. Finally, cells were eluted by glacial acetic acid and quantified at 570 nm wavelength on a microplate reader (Corning Inc., NY, USA).

### Statistical analysis

All data are expressed as means ± standard error of means (SEMs), and obtained from multiple independent experiments (at least triple repeats) after being processed on a Graphic Prism software. The two tailed *t*-test and one-way ANOVA analysis were utilized to evaluate the differences between groups. *p* < 0.05 was considered statistically significant.

## Results

### BIRC5 was upregulated in PC progression

**Fig. 1:**
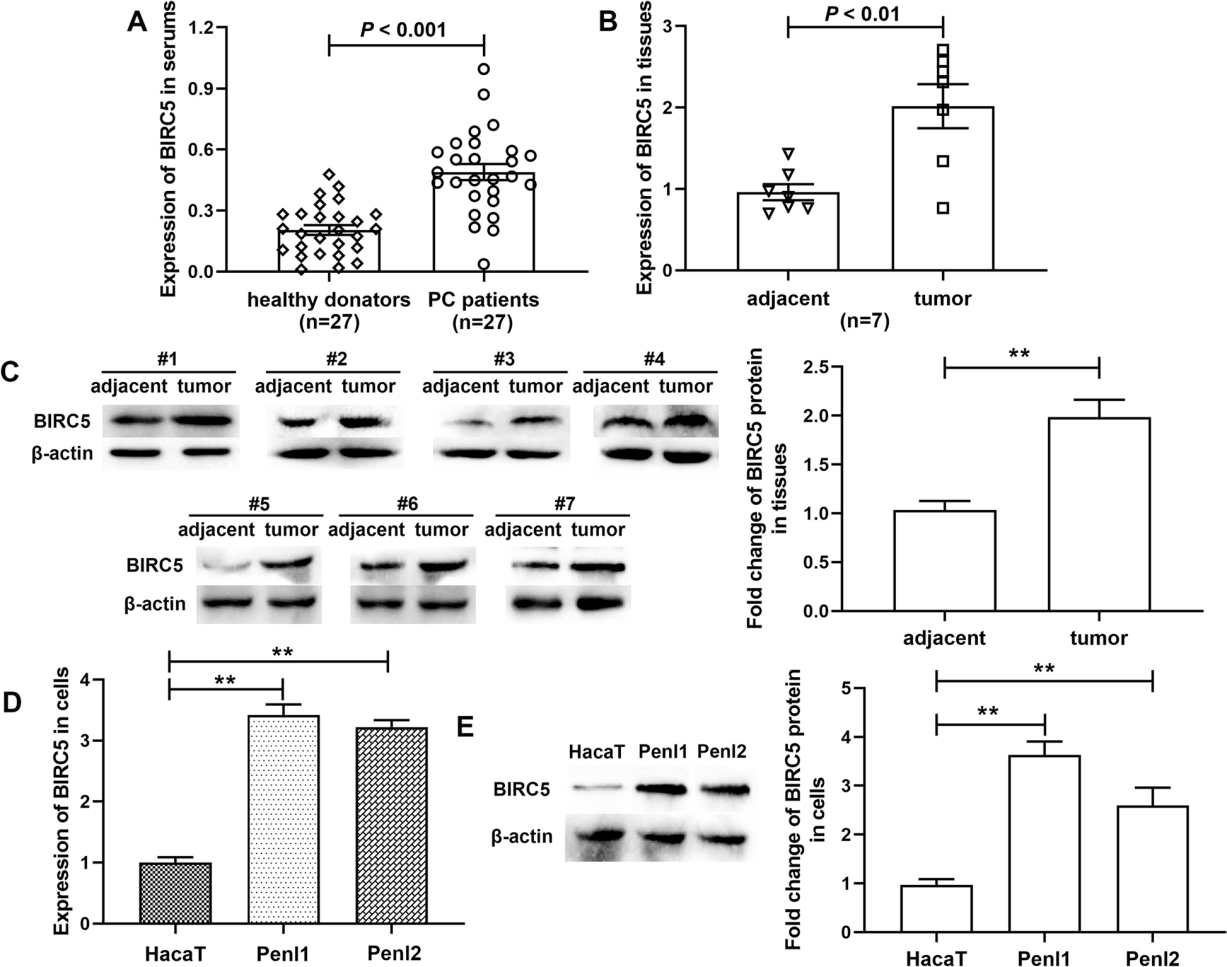
BIRC5 was upregulated during PC progression. BIRC5 mRNA expressions in serum (**A**) and tissues (**B**) of PC cases, as well as PC cells (Penl1 and Penl2) (**D**), were detected by RT-qPCR. ^**^ stands for *p* < 0.01 vs HacaT cells (**D**). (**C** and **E**) BIRC5 protein expression in PC tissues (**C**) and cells (**E**) was investigated using western blotting. The columns were presented as the mean ± SEM (*n* ≥ 3)

In our study, the expression profile of BIRC5 in PC development was evaluated at first. According to Fig. 1A, the mRNA level of BIRC5 in the serum of PC patients was significantly elevated compared to that in healthy donors. We also discovered that BIRC5 mRNA (Fig. 1B) and protein (Fig. 1C) expressions were dramatically upregulated in PC tissues of patients compared with adjacent tissues. Moreover, both Penl1 and Penl2 cells showed dramatically elevated BIRC5 expression than HaCaT cells, with regard to mRNA and protein levels (Fig. 1D and E).

### Silencing BIRC5 inhibited the growth and motility of PC cells

**Fig. 2:**
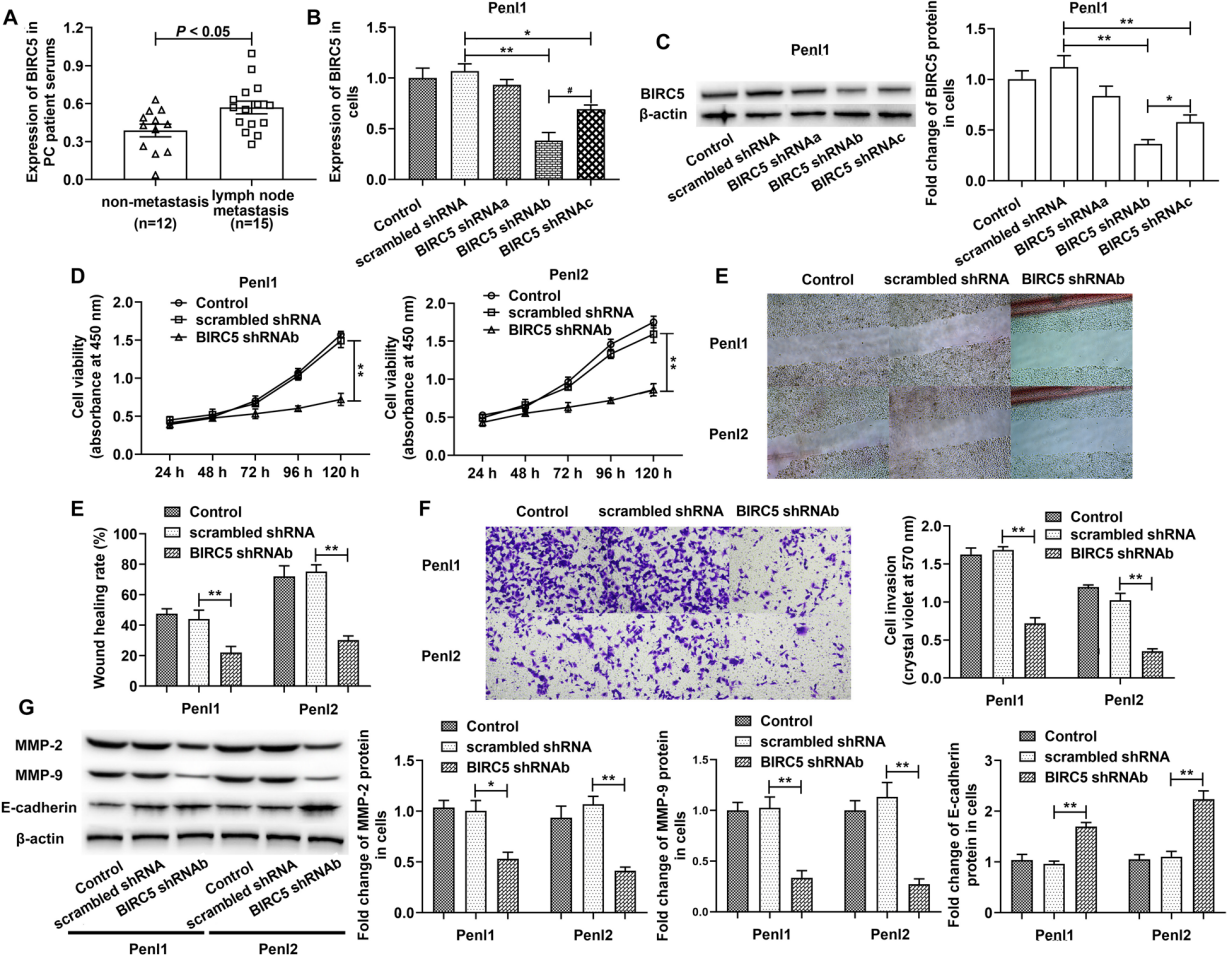
Silencing BIRC5 inhibited cell viability, migration, invasion of PC cells. (**A**) Expression of BIRC5 was detected using RT-qPCR. PC cells were transfected with shRNA lentiviral vectors. (**B** and **C**) The silencing efficiency of BIRC5 shRNAs was confirmed by RT-qPCR and western blot. (**D**) Cell viability, migration and invasion were studied by CCK-8, wound healing and Transwell assays. (**G**) Western blotting presented the expressions of target EMT proteins. The columns were presented as the mean ± SEM (*n* ≥ 3). ^*^ and ^**^ stood for *p* < 0.05 and *p* < 0.01

As shown in Fig. 2A, BIRC5 expression in the serum of PC patients who suffered lymph node metastasis was dramatically higher than those in the non-metastasis group. Next, we performed a loss-of-function assay to investigate the effect of BIRC5 on the development of PC. The efficiency analysis confirmed that shRNAb could effectively block the mRNA and protein expressions of BIRC (Fig. 2B and C). As expected, silencing BIRC5 inhibited PC cell growth (Fig. 2D). Migration and invasion abilities of both Penl1 and Penl2 cells were also depressed by the transfection of BIRC5 shRNAb (Fig. 2E and F). Besides, protein levels of MMP2 and MMP9 were significantly inhibited by BIRC5 silencing while E-cadherin expression was promoted (Fig. 2G). The evidence suggested that BIRC5 suppression attenuated the growth and motility of PC cells.

### Silencing BIRC5 alleviated IFN-γ-induced aggravation of PC development

**Fig. 3:**
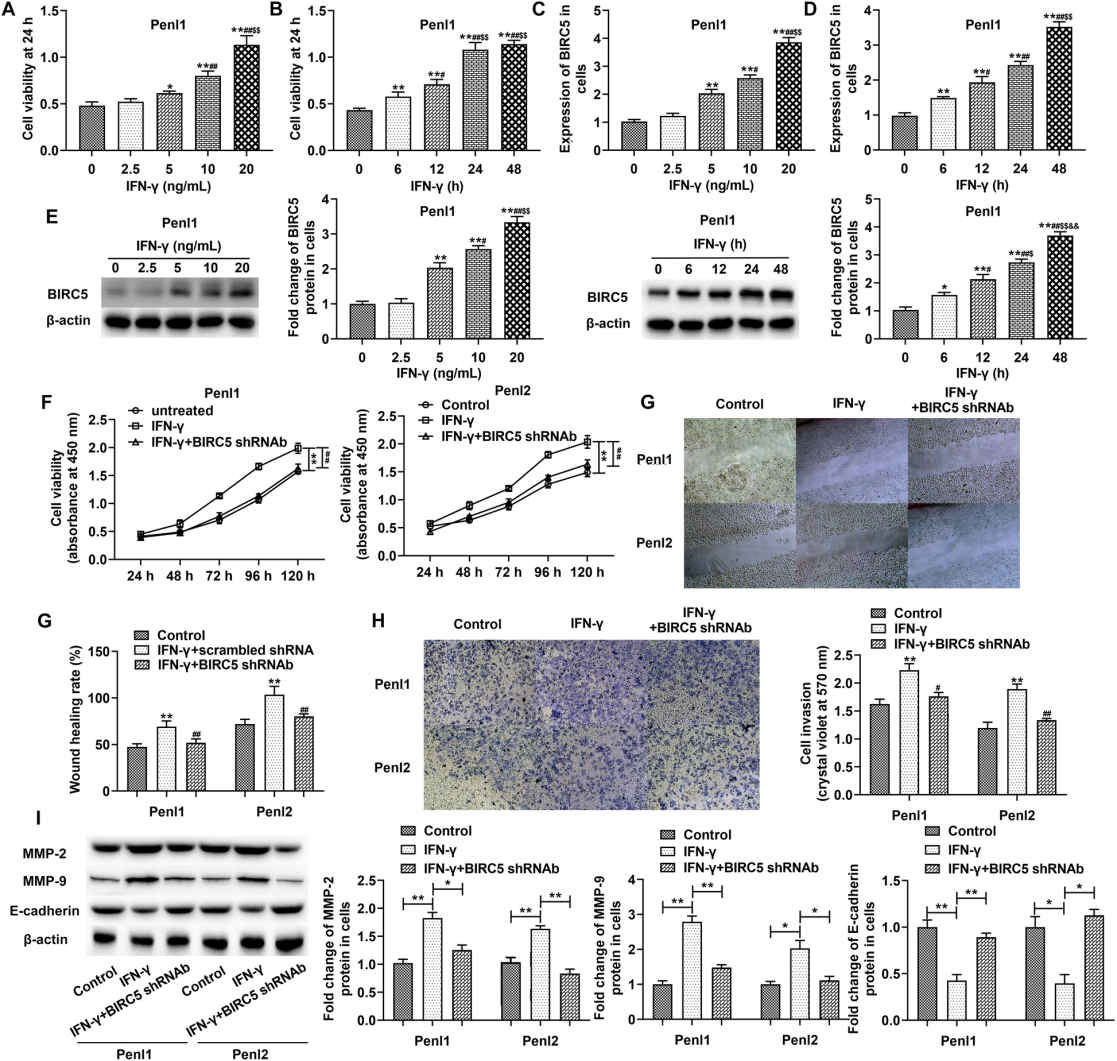
Silencing BIRC5 depressed IFN-γ-induced aggravation of PC cell processes. PC cells were transfected with BIRC shRNAb lentiviral vector before IFN-γ treatment (20 ng/mL, 24 h). (**A** and **B**) CCK-8 assay detected cell viability. (**C**, **D** and **E**) RT-qPCR and western blot assays were performed to measure the expression of BIRC5. In **A** and **C**, * *p* < 0.05 and ** *p* < 0.01 versus 2.5 ng/mL group, ^#^ *p* < 0.05 and ^##^ *p* < 0.01 versus 5 ng/mL group, ^$$^ *p* < 0.01 versus 10 ng/mL; in **B**, **D** and **E**, ** *p* < 0.01 versus 0 h, ^#^ *p* < 0.05 and ^##^ *p* < 0.01 versus 6 h, ^$$^ *p* < 0.01 versus 12 h and 24 h respectively. (**F**, **G** and **H**) Cell viability, migration and invasion were evaluated using CCK-8, wound healing and Transwell methods. (**I)** The expression of EMT proteins were measured by western blot assay. The columns were presented as the mean ± SEM (*n* ≥ 3). ^*^ *p* < 0.05 and ^**^ *p* < 0.01

To explore the effect of inflammation on PC development, we treated PC cells with IFN-γ, a multifunctional pro-inflammatory cytokine. As the results shown, IFN-γ dramatically enhanced the viability of PC cells in a dose dependent manner and a time dependent manner except that there was no significance between 24 and 48 h (Fig. 3A and B). The expression of BIRC5 was also upregulated by IFN-γ stimulation (Fig. 3C and D). Moreover, PC cell viability in the BIRC5 knockdown associated with IFN-γ group was dramatically decreased compared with the IFN-γ group (Fig. 3E). The cell migrated and invaded abilities, as well as EMT process related protein expressions (MMP2 and MMP9), of Penl1 and Penl2 cells were significantly elevated which were decreased by BIRC5 silencing; and the depressed expression of E-cadherin was reversed in the BIRC5 knockdown group (Fig. 3F, G, H and I).

### BIRC5 knockdown inhibited tumor development and IFN-γ-induced PC tumor aggravation *in vivo*

**Fig. 4:**
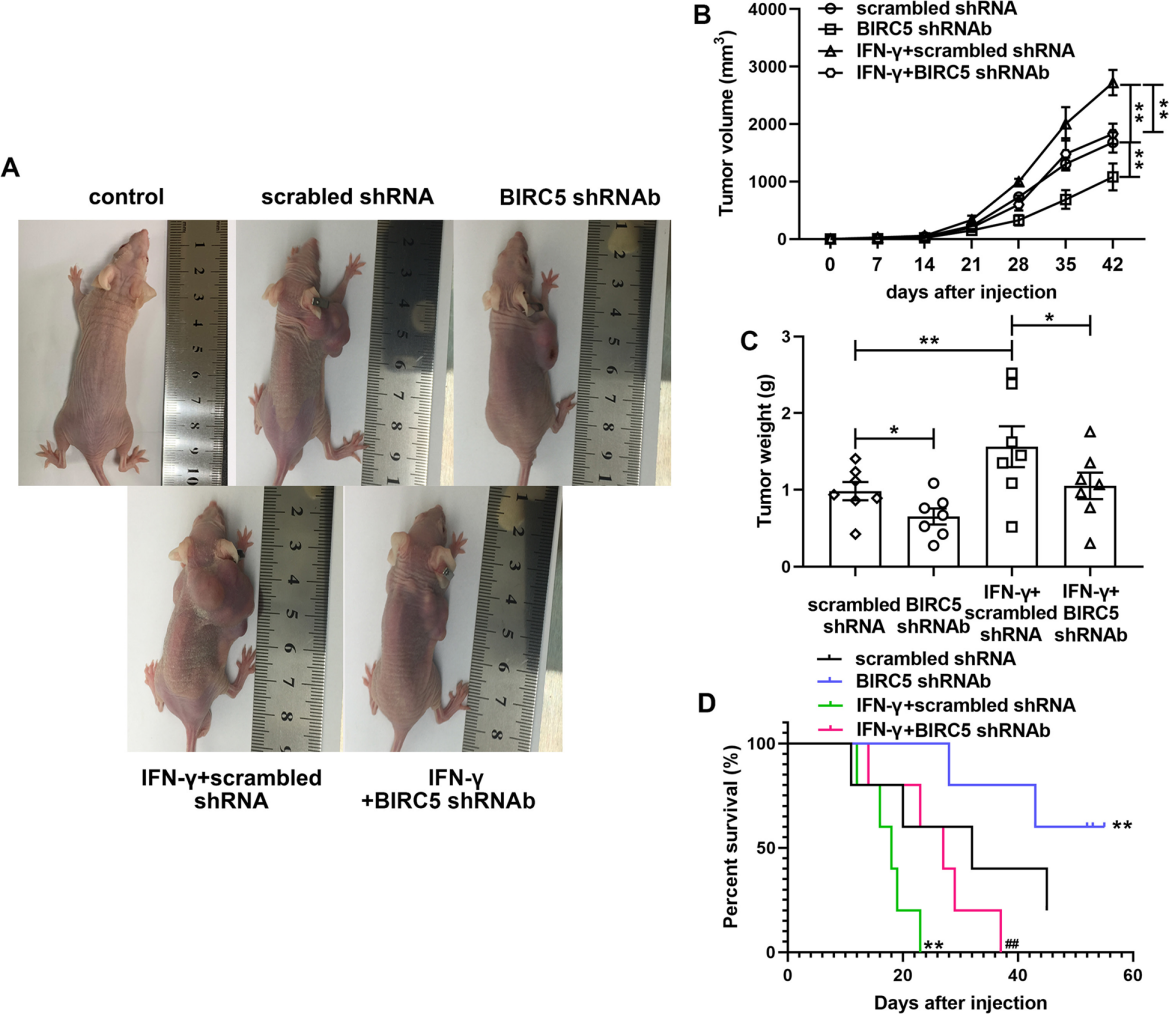
Silencing BIRC5 depressed the tumor growth and survival of PC xenograft mice. Penl1 cells (100 µL containing 1 × 10^6^ cells) in different groups (scrambled shRNA, BIRC5 shRNAb, IFN-γ + scrambled shRNA; IFN-γ + BIRC5 shRNAb) and HacaT cells (control) were subcutaneously inoculated at the right axilla after mice were anesthetized. **A** Image of tumor growth in living mice. (**B**, **C** and **D**) Tumor volume and tumor weight were measured and survival rate was calculated. The columns were presented as the mean ± SEM. ^*^ and ^**^ stands for *p* < 0.05 and *p* < 0.01 compared with scrambled shRNA group. ^##^ stands for *p* < 0.05 compared with IFN-γ + scrambled shRNA group. *n* = 5 in every group

We next validated the role of BIRC5 in PC development under an IMT condition. The morphological size, tumor volume and weight in the BIRC5 silencing group were significantly lower than those in the model group; IFN-γ dramatically increased these indexes of PC xenograft tumor in mice while BIRC5 also weaken the effect of IFN-γ *in vivo* (Fig. 4A, B and C). Moreover, BIRC5 knockdown obviously improved the survival of PC mice compared with model group; IFN-γ accelerated the death of mice while BIRC5 silencing prolonged the survival time of PC mice those under IFN-γ stimulation (Fig. 4D).

## Discussion

The relationship between cell processes and BIRC5 function has attracted more and more attention in the field of human physiological development and medical research. Scientists discovered that the high activation of BIRC5 depressed the respiration of mitochondria and induced its fragmentation, finally led to Foxo3-dependent cell apoptosis by preventing reactive oxygen species accumulation, in neuroblastoma [27]. Gil-Kulik et al*.* indicated that BIRC5 was a pivotal mediator of cellular mitosis and maintained differentiation of stem cells in humans [28]. Moreover, emerging studies had reported that BIRC5 had an abnormally increased expression in diverse cancer tissues and played a critical role in the malignant progression of tumors [13]. A high expression level of BIRC5 has been identified in lung adenocarcinoma and was associated with high risk of distant metastasis and tumor bearing in patients [29]. Kimia and colleagues uncovered a significant correlation between the increased copy number of BIRC5 and breast cancer individuals [30]. Moreover, the molecular data generation research of Marchi et al*.* indicated that BIRC5 overexpression was closely associated with poor survival of PC patients [31]. Our study confirmed the aberrant upregulation of BIRC5 in PC serum and tissues, as well as cell lines, which may suggest BIRC5 as a prognostic biomarker for PC.

Furthermore, the present research demonstrated that BIRC5 silencing dramatically suppressed cell proliferation, migration and invasion, as well as tumor growth *in vivo*, in parallelly. Similar to our findings, the oncogenic role of BIRC5 has been unveiled in other tumor diseases. For instance, Marina et al*.* found that the increased BIRC5 level in U251-MG cells (glioma cells) led to deteriorative DNA damage and structural chromosomal aberrations, promoting cell proliferation and decreasing cell apoptosis [14]. Blocking BIRC5 expression by using YM155 inhibitor effectively reduced the migration and invasion rates of ovarian cancer cells, EMT, migration and invasion were also inhibited [32]. Except that, downregulating BIRC5 improved the efficiency of anti-myeloma drugs and triggered cell apoptosis, thereby developing the therapeutic benefit for multiple myeloma [33].

Emerging studies have revealed that prolonged inflammation was a severe factor that induced tumor initiation and malignancy [34]. Cell metabolism and homeostasis were interrupted under persistent inflammatory condition leading to the aggressive growth of tumor [21]. As well known, tumor microenvironment was the closest growth environment of tumor cells and provided the basement for neoplasm evolution [35]. Besides, the inflammatory cytokines secreted by immune cells that were recruited into the tumor microenvironment were validated to induce uncontrollable cell proliferation and death resistance [36]. These suggested that alterations in inflammation occurring in the tumor microenvironment had an important linkage with the development of cancer diseases. Cell viability, invasion and EMT processes of colorectal cancer cells were promoted in LPS-induced inflammatory condition [37]. Another evidence presented that IFN-γ stimulated the tumor growth and metastasis of gastric tumor in its xenograft model [38]. Consistently, our study indicated that IFN-γ dramatically promoted the proliferation, migration and invasion of PC cells, as well as tumor growth *in vivo*, while BIRC5 knockdown reversed these effects.

## Conclusions

We identified that BIRC5 was upregulated in PC tissues and cell lines. Silencing BIRC5 inhibited the proliferation, migration and invasion of PC cells. Moreover, the ITM aggravated PC progression which was attenuated by BIRC5 depression. The effect of BIRC5 on tumor growth was also verified in PC xenograft model mice. Our study provided BIRC5 as a potential diagnostic and therapeutic target for PC.

## Supplementary Information

**Additional file 1:** **Fig. S1.** Silencing BIRC5 inhibited cell viability, migration, invasion of PC cells under IFN-γ treatment (20 ng/mL, 24 h).PC cellswere transfected with shRNA lentiviral vectors. (A and B) Cell viability was studied by CCK-8 assay. (C and D) Wound healing assay and Transwell assay were performed to detect cell migration and invasion. The columns were presented as the mean ± SEM (*n* ≥ 3). ^*^ and^**^stood for*p* 0.05 and *p* 0.01. **Fig. S2.** Silencing BIRC5 depressed the aggravated tumor growth and survival of PC xenograft mice induced by IFN-γ.Penl1 cells in different groups (IFN-γ; IFN-γ + BIRC5 shRNAb) and untreated Penl1 cells (model) were subcutaneously inoculated at the right axilla (100 µL containing 1 × 10^6^ cells) after mice were anesthetized. (A) Image of tumor growth in living mice. (B, C and D) Tumor volume and tumor weight were measured and survival rate was calculated. The columns were presented as the mean ± SEM. ^*^^*^ stands for *p* 0.01 compared with model group. ^##^ stands for *p* 0.01 compared with IFN-γ group. *n*= 5 in every group.

## Data Availability

Data used and analyzed during the current study are not publicly available due to the restriction of the progressions of foundation item and research, but are available from the corresponding author on reasonable request.

## Abbreviations

BIRC5: Baculoviral IAP Repeat Containing 5
DMEM: Dulbecco’s modified Eagle’s medium
ITM: Inflammatory tumor microenvironment
IAPs: Inhibitor of apoptosis proteins
IFN-γ: Interferon
MMP2: Matrix Metalloproteinase 2
OD: Optical density
PC: Penile carcinoma
SEM: Standard error of mean
shRNAs: Short hairpin RNAs
