# Supplementary figures and images for: BIRC5 regulates inflammatory tumor microenvironment-induced aggravation of penile cancer development in vitro and in vivo
Source: BMC Cancer. 2022 Apr 23;22:448. doi: 10.1186/s12885-022-09500-9 (PMC9035256; doi:10.1186/s12885-022-09500-9)

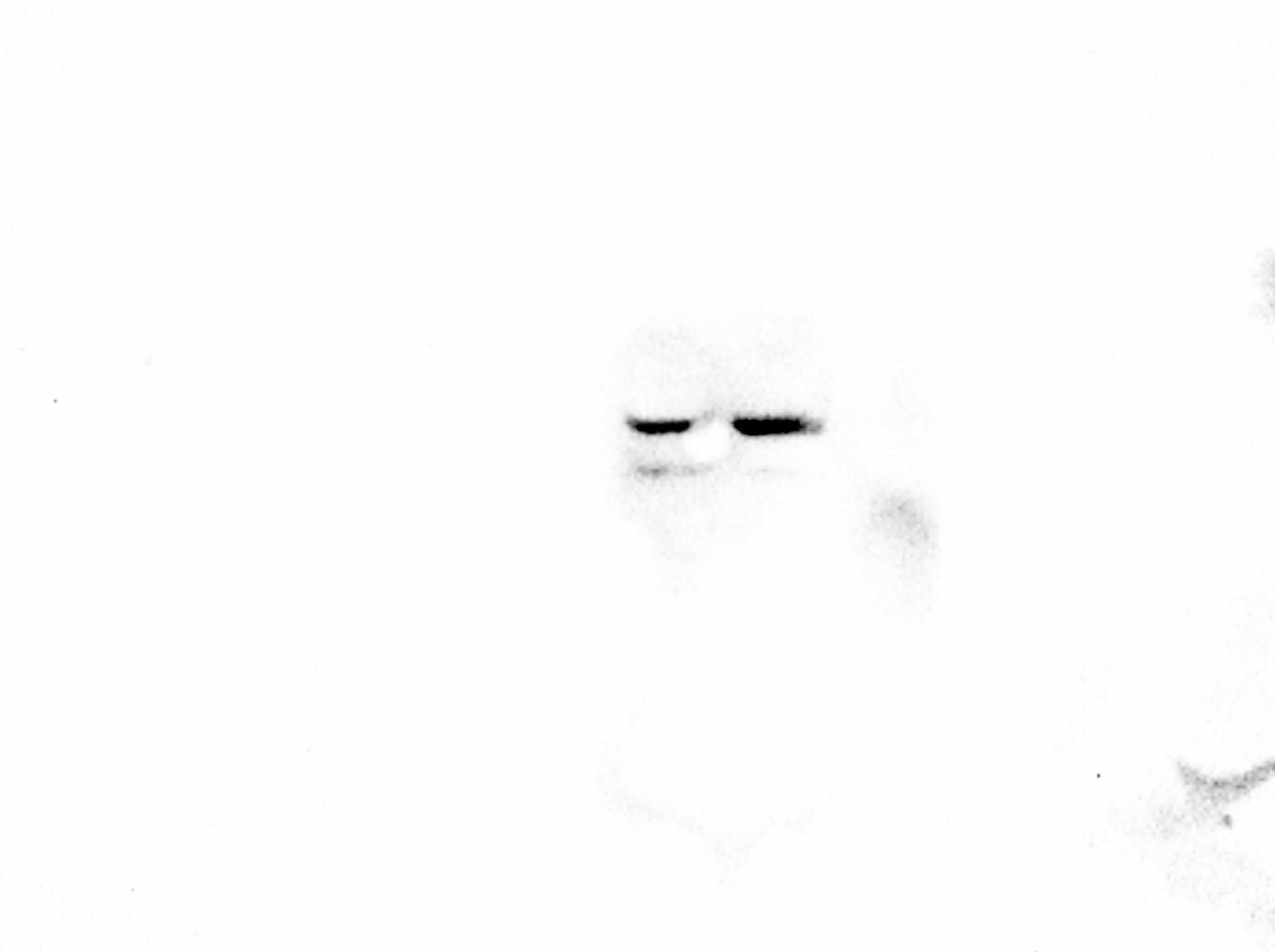

Supplement: Supplementary file 1 — Additional file 1: Fig. S1. Silencing BIRC5 inhibited cell viability, migration, invasion of PC cells under IFN-γ treatment (20 ng/mL, 24 h).PC cellswere transfected with shRNA lentiviral vectors. (A and B) Cell viability was studied by CCK-8 assay. (C and D) Wound healing assay and Transwell assay were performed to detect cell migration and invasion. The columns were presented as the mean ± SEM (n ≥ 3). * and**stood forp 0.05 and p 0.01. Fig. S2. Silencing BIRC5 depressed the aggravated tumor growth and survival of PC xenograft mice induced by IFN-γ.Penl1 cells in different groups (IFN-γ; IFN-γ + BIRC5 shRNAb) and untreated Penl1 cells (model) were subcutaneously inoculated at the right axilla (100 µL containing 1 × 10^6^ cells) after mice were anesthetized. (A) Image of tumor growth in living mice. (B, C and D) Tumor volume and tumor weight were measured and survival rate was calculated. The columns were presented as the mean ± SEM. ** stands for p 0.01 compared with model group. ## stands for p 0.01 compared with IFN-γ group. n= 5 in every group. [file 12885_2022_9500_MOESM1_ESM.zip › Supplementary/1-Fig.1C BIRC5-2.jpg]

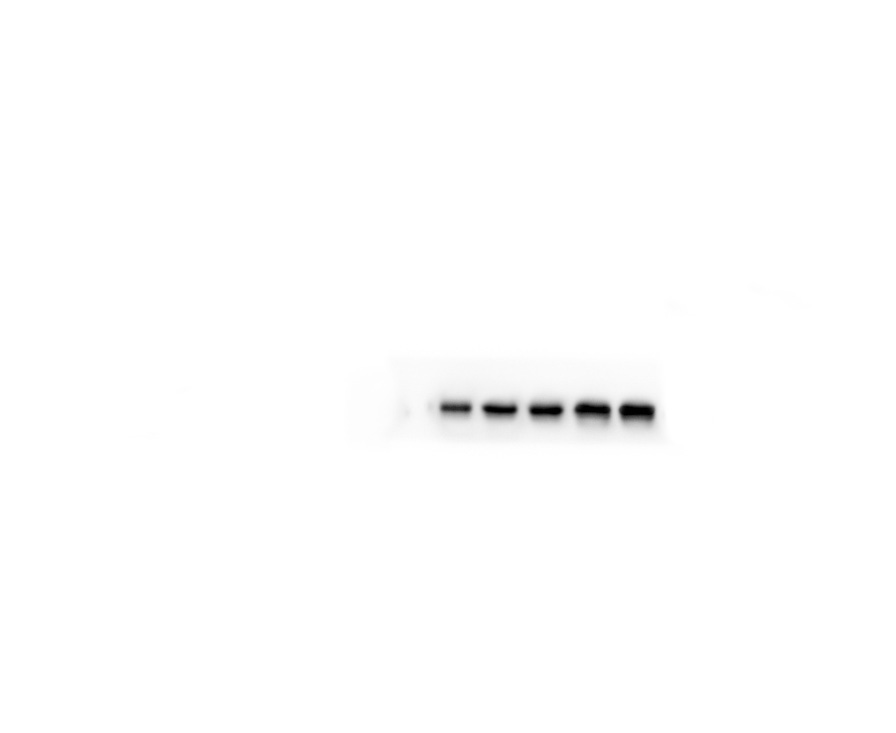

Supplement: Supplementary file 1 — Additional file 1: Fig. S1. Silencing BIRC5 inhibited cell viability, migration, invasion of PC cells under IFN-γ treatment (20 ng/mL, 24 h).PC cellswere transfected with shRNA lentiviral vectors. (A and B) Cell viability was studied by CCK-8 assay. (C and D) Wound healing assay and Transwell assay were performed to detect cell migration and invasion. The columns were presented as the mean ± SEM (n ≥ 3). * and**stood forp 0.05 and p 0.01. Fig. S2. Silencing BIRC5 depressed the aggravated tumor growth and survival of PC xenograft mice induced by IFN-γ.Penl1 cells in different groups (IFN-γ; IFN-γ + BIRC5 shRNAb) and untreated Penl1 cells (model) were subcutaneously inoculated at the right axilla (100 µL containing 1 × 10^6^ cells) after mice were anesthetized. (A) Image of tumor growth in living mice. (B, C and D) Tumor volume and tumor weight were measured and survival rate was calculated. The columns were presented as the mean ± SEM. ** stands for p 0.01 compared with model group. ## stands for p 0.01 compared with IFN-γ group. n= 5 in every group. [file 12885_2022_9500_MOESM1_ESM.zip › Supplementary/1-Fig.3E BIRC5.jpg]

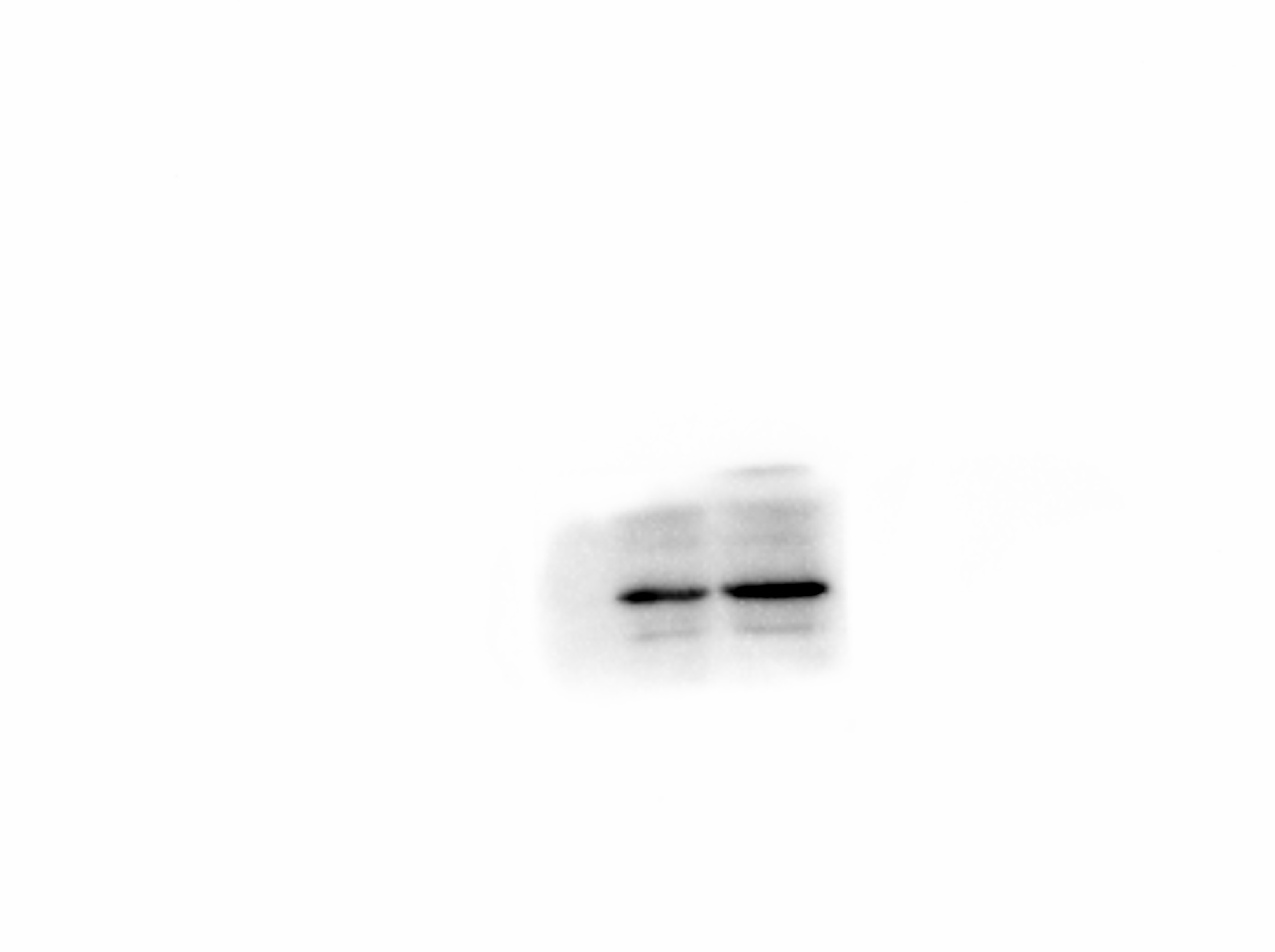

Supplement: Supplementary file 1 — Additional file 1: Fig. S1. Silencing BIRC5 inhibited cell viability, migration, invasion of PC cells under IFN-γ treatment (20 ng/mL, 24 h).PC cellswere transfected with shRNA lentiviral vectors. (A and B) Cell viability was studied by CCK-8 assay. (C and D) Wound healing assay and Transwell assay were performed to detect cell migration and invasion. The columns were presented as the mean ± SEM (n ≥ 3). * and**stood forp 0.05 and p 0.01. Fig. S2. Silencing BIRC5 depressed the aggravated tumor growth and survival of PC xenograft mice induced by IFN-γ.Penl1 cells in different groups (IFN-γ; IFN-γ + BIRC5 shRNAb) and untreated Penl1 cells (model) were subcutaneously inoculated at the right axilla (100 µL containing 1 × 10^6^ cells) after mice were anesthetized. (A) Image of tumor growth in living mice. (B, C and D) Tumor volume and tumor weight were measured and survival rate was calculated. The columns were presented as the mean ± SEM. ** stands for p 0.01 compared with model group. ## stands for p 0.01 compared with IFN-γ group. n= 5 in every group. [file 12885_2022_9500_MOESM1_ESM.zip › Supplementary/Fig.1C BIRC5-1.jpg]

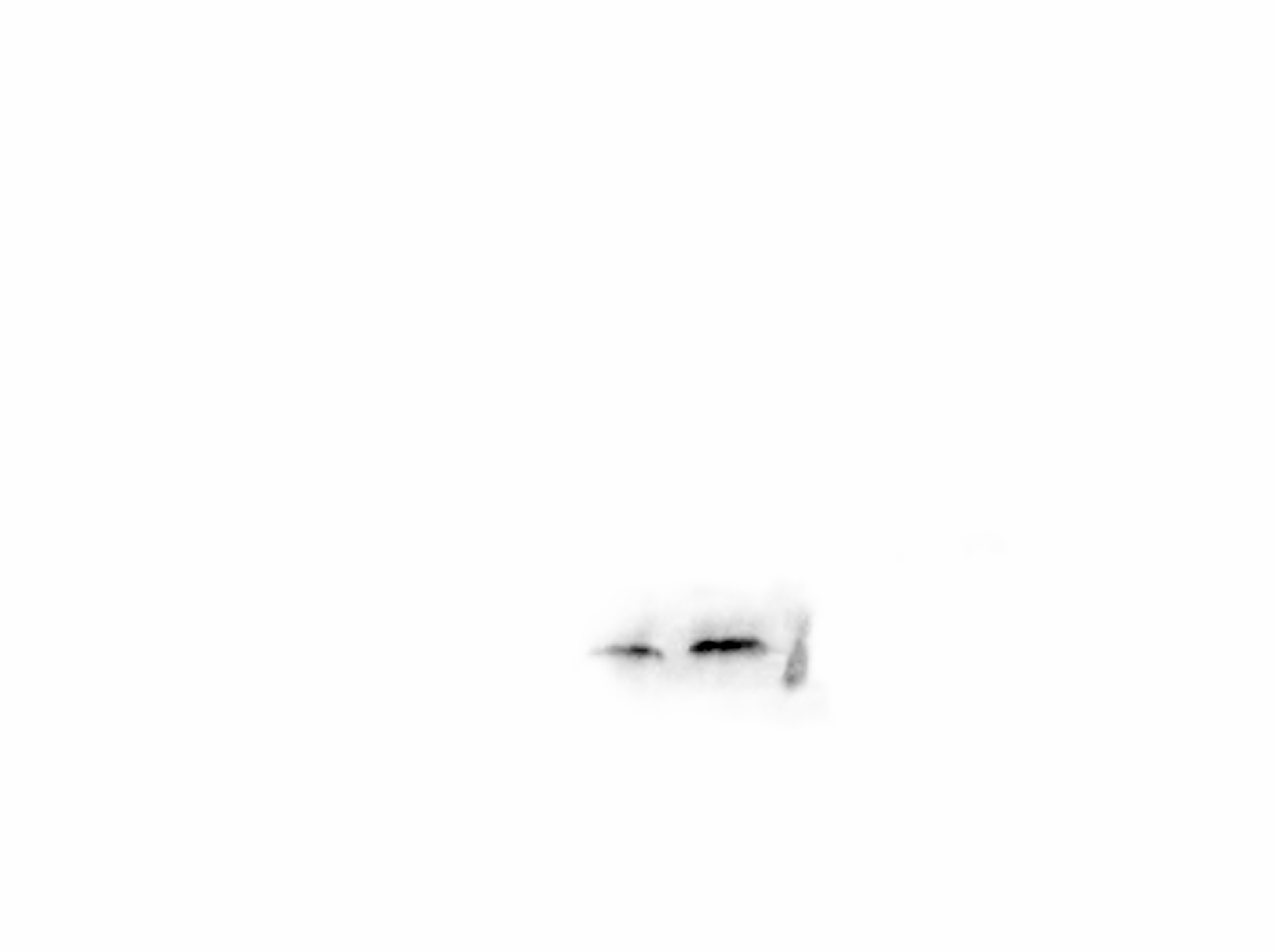

Supplement: Supplementary file 1 — Additional file 1: Fig. S1. Silencing BIRC5 inhibited cell viability, migration, invasion of PC cells under IFN-γ treatment (20 ng/mL, 24 h).PC cellswere transfected with shRNA lentiviral vectors. (A and B) Cell viability was studied by CCK-8 assay. (C and D) Wound healing assay and Transwell assay were performed to detect cell migration and invasion. The columns were presented as the mean ± SEM (n ≥ 3). * and**stood forp 0.05 and p 0.01. Fig. S2. Silencing BIRC5 depressed the aggravated tumor growth and survival of PC xenograft mice induced by IFN-γ.Penl1 cells in different groups (IFN-γ; IFN-γ + BIRC5 shRNAb) and untreated Penl1 cells (model) were subcutaneously inoculated at the right axilla (100 µL containing 1 × 10^6^ cells) after mice were anesthetized. (A) Image of tumor growth in living mice. (B, C and D) Tumor volume and tumor weight were measured and survival rate was calculated. The columns were presented as the mean ± SEM. ** stands for p 0.01 compared with model group. ## stands for p 0.01 compared with IFN-γ group. n= 5 in every group. [file 12885_2022_9500_MOESM1_ESM.zip › Supplementary/Fig.1C BIRC5-3.jpg]

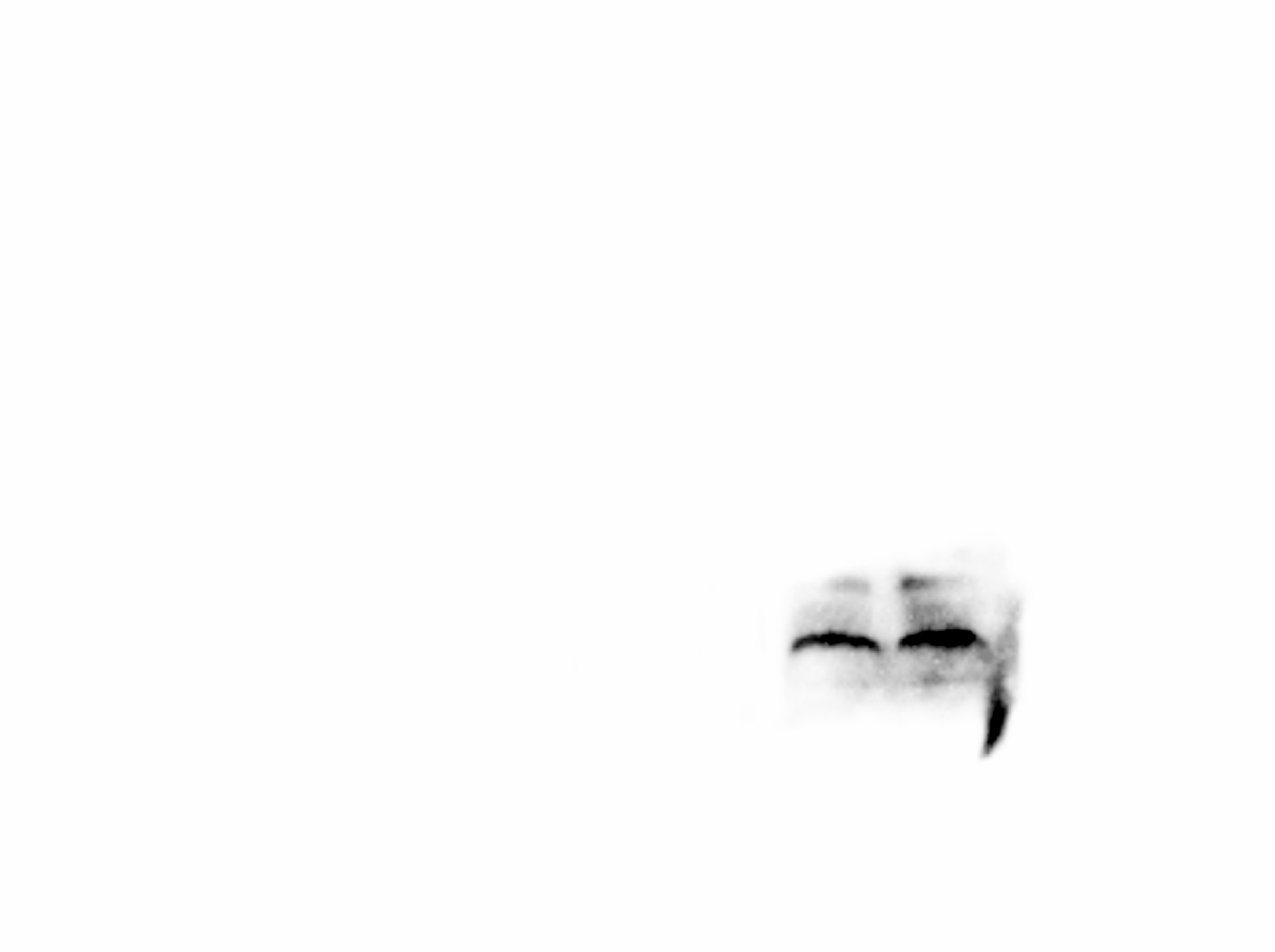

Supplement: Supplementary file 1 — Additional file 1: Fig. S1. Silencing BIRC5 inhibited cell viability, migration, invasion of PC cells under IFN-γ treatment (20 ng/mL, 24 h).PC cellswere transfected with shRNA lentiviral vectors. (A and B) Cell viability was studied by CCK-8 assay. (C and D) Wound healing assay and Transwell assay were performed to detect cell migration and invasion. The columns were presented as the mean ± SEM (n ≥ 3). * and**stood forp 0.05 and p 0.01. Fig. S2. Silencing BIRC5 depressed the aggravated tumor growth and survival of PC xenograft mice induced by IFN-γ.Penl1 cells in different groups (IFN-γ; IFN-γ + BIRC5 shRNAb) and untreated Penl1 cells (model) were subcutaneously inoculated at the right axilla (100 µL containing 1 × 10^6^ cells) after mice were anesthetized. (A) Image of tumor growth in living mice. (B, C and D) Tumor volume and tumor weight were measured and survival rate was calculated. The columns were presented as the mean ± SEM. ** stands for p 0.01 compared with model group. ## stands for p 0.01 compared with IFN-γ group. n= 5 in every group. [file 12885_2022_9500_MOESM1_ESM.zip › Supplementary/Fig.1C BIRC5-4.jpg]

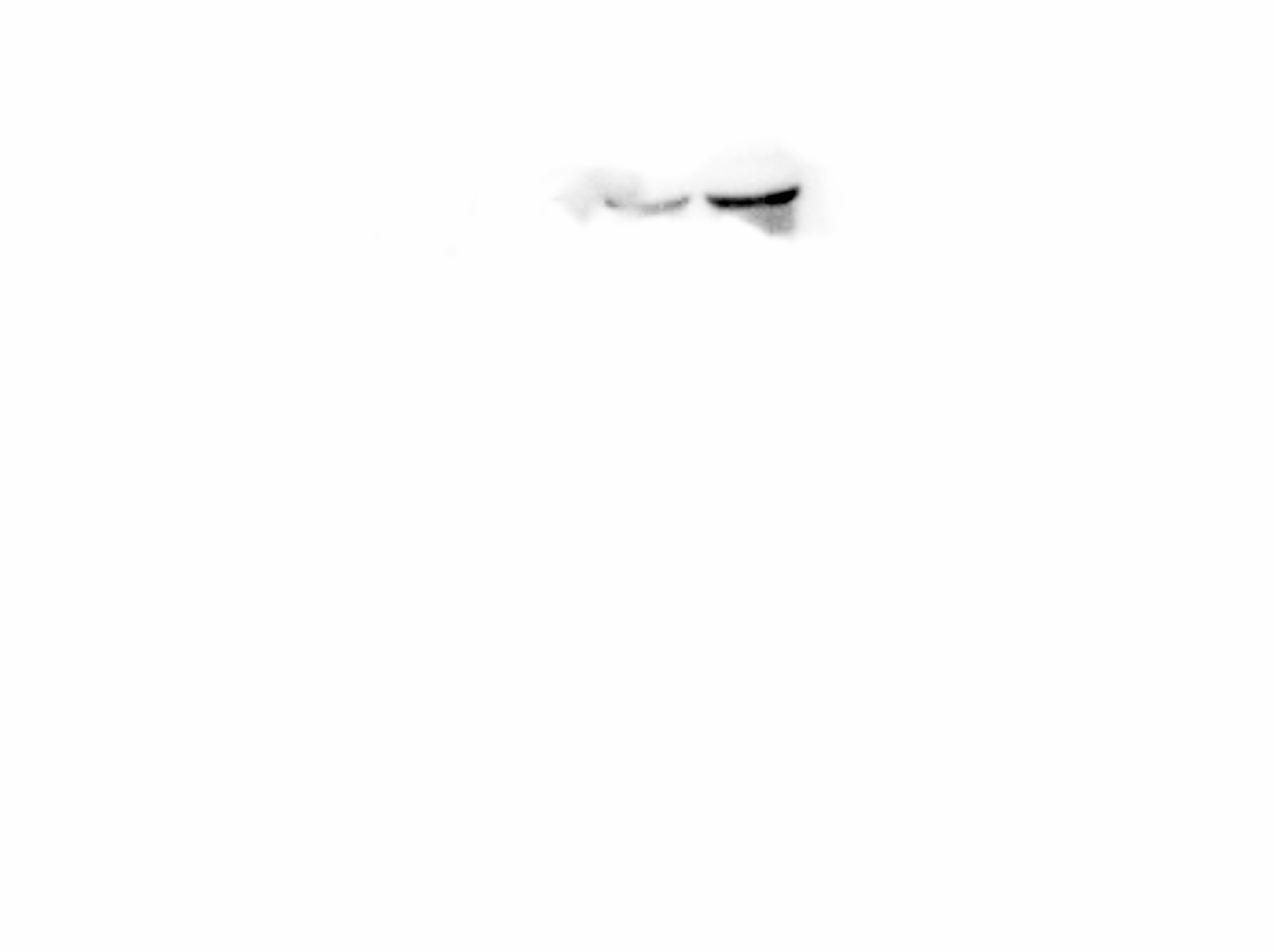

Supplement: Supplementary file 1 — Additional file 1: Fig. S1. Silencing BIRC5 inhibited cell viability, migration, invasion of PC cells under IFN-γ treatment (20 ng/mL, 24 h).PC cellswere transfected with shRNA lentiviral vectors. (A and B) Cell viability was studied by CCK-8 assay. (C and D) Wound healing assay and Transwell assay were performed to detect cell migration and invasion. The columns were presented as the mean ± SEM (n ≥ 3). * and**stood forp 0.05 and p 0.01. Fig. S2. Silencing BIRC5 depressed the aggravated tumor growth and survival of PC xenograft mice induced by IFN-γ.Penl1 cells in different groups (IFN-γ; IFN-γ + BIRC5 shRNAb) and untreated Penl1 cells (model) were subcutaneously inoculated at the right axilla (100 µL containing 1 × 10^6^ cells) after mice were anesthetized. (A) Image of tumor growth in living mice. (B, C and D) Tumor volume and tumor weight were measured and survival rate was calculated. The columns were presented as the mean ± SEM. ** stands for p 0.01 compared with model group. ## stands for p 0.01 compared with IFN-γ group. n= 5 in every group. [file 12885_2022_9500_MOESM1_ESM.zip › Supplementary/Fig.1C BIRC5-5.jpg]

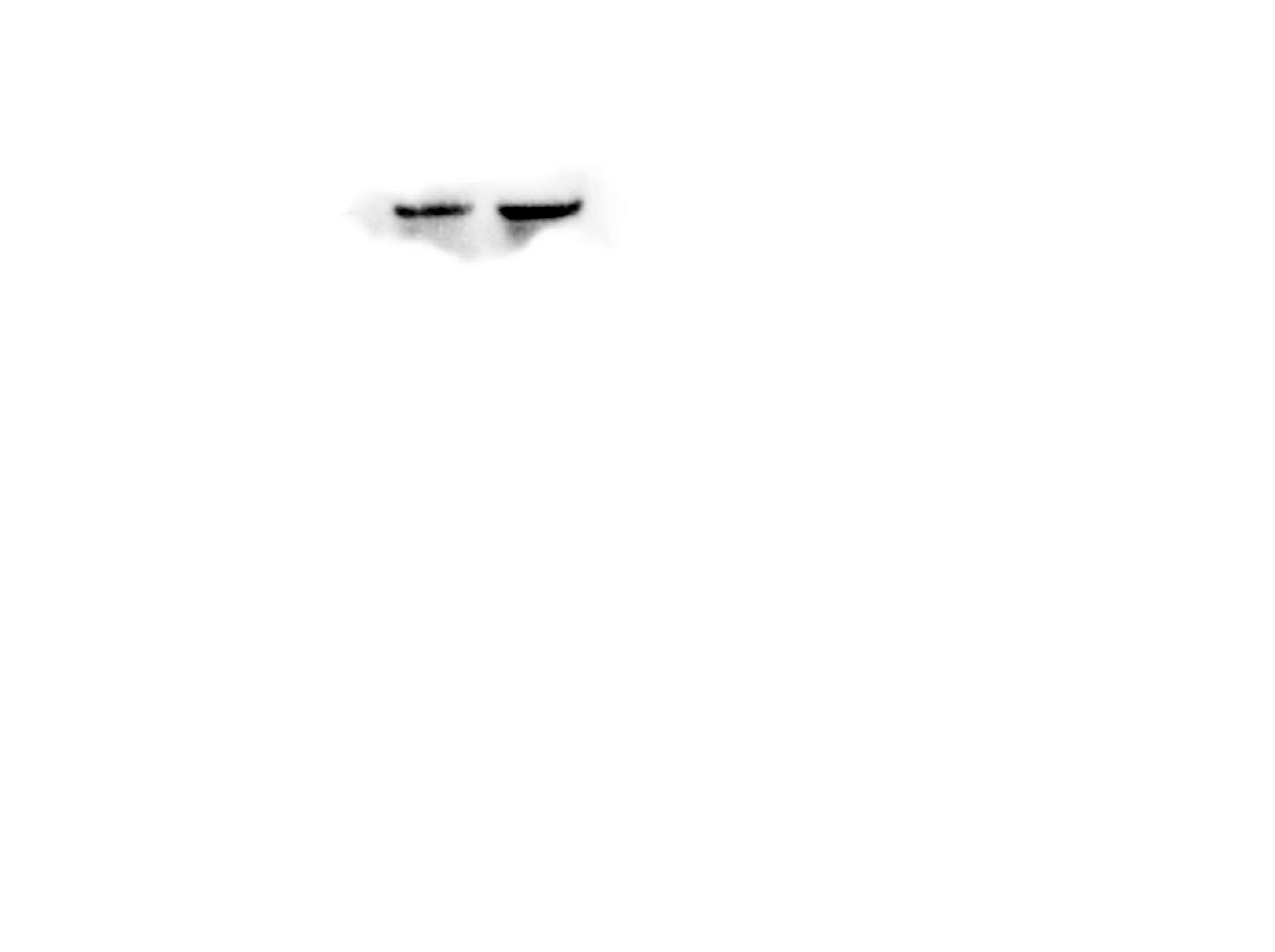

Supplement: Supplementary file 1 — Additional file 1: Fig. S1. Silencing BIRC5 inhibited cell viability, migration, invasion of PC cells under IFN-γ treatment (20 ng/mL, 24 h).PC cellswere transfected with shRNA lentiviral vectors. (A and B) Cell viability was studied by CCK-8 assay. (C and D) Wound healing assay and Transwell assay were performed to detect cell migration and invasion. The columns were presented as the mean ± SEM (n ≥ 3). * and**stood forp 0.05 and p 0.01. Fig. S2. Silencing BIRC5 depressed the aggravated tumor growth and survival of PC xenograft mice induced by IFN-γ.Penl1 cells in different groups (IFN-γ; IFN-γ + BIRC5 shRNAb) and untreated Penl1 cells (model) were subcutaneously inoculated at the right axilla (100 µL containing 1 × 10^6^ cells) after mice were anesthetized. (A) Image of tumor growth in living mice. (B, C and D) Tumor volume and tumor weight were measured and survival rate was calculated. The columns were presented as the mean ± SEM. ** stands for p 0.01 compared with model group. ## stands for p 0.01 compared with IFN-γ group. n= 5 in every group. [file 12885_2022_9500_MOESM1_ESM.zip › Supplementary/Fig.1C BIRC5-6.jpg]

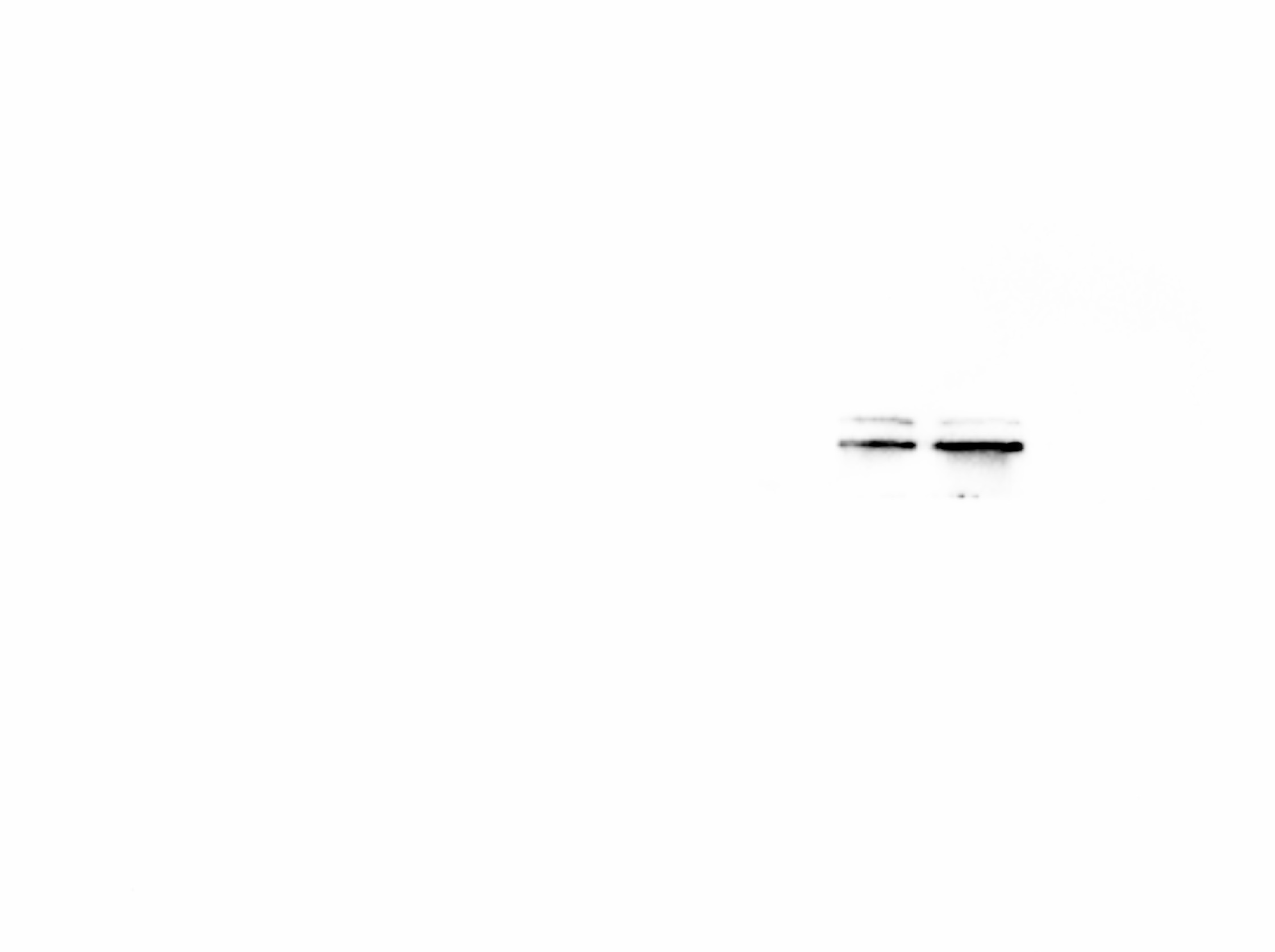

Supplement: Supplementary file 1 — Additional file 1: Fig. S1. Silencing BIRC5 inhibited cell viability, migration, invasion of PC cells under IFN-γ treatment (20 ng/mL, 24 h).PC cellswere transfected with shRNA lentiviral vectors. (A and B) Cell viability was studied by CCK-8 assay. (C and D) Wound healing assay and Transwell assay were performed to detect cell migration and invasion. The columns were presented as the mean ± SEM (n ≥ 3). * and**stood forp 0.05 and p 0.01. Fig. S2. Silencing BIRC5 depressed the aggravated tumor growth and survival of PC xenograft mice induced by IFN-γ.Penl1 cells in different groups (IFN-γ; IFN-γ + BIRC5 shRNAb) and untreated Penl1 cells (model) were subcutaneously inoculated at the right axilla (100 µL containing 1 × 10^6^ cells) after mice were anesthetized. (A) Image of tumor growth in living mice. (B, C and D) Tumor volume and tumor weight were measured and survival rate was calculated. The columns were presented as the mean ± SEM. ** stands for p 0.01 compared with model group. ## stands for p 0.01 compared with IFN-γ group. n= 5 in every group. [file 12885_2022_9500_MOESM1_ESM.zip › Supplementary/Fig.1C BIRC5-7.jpg]

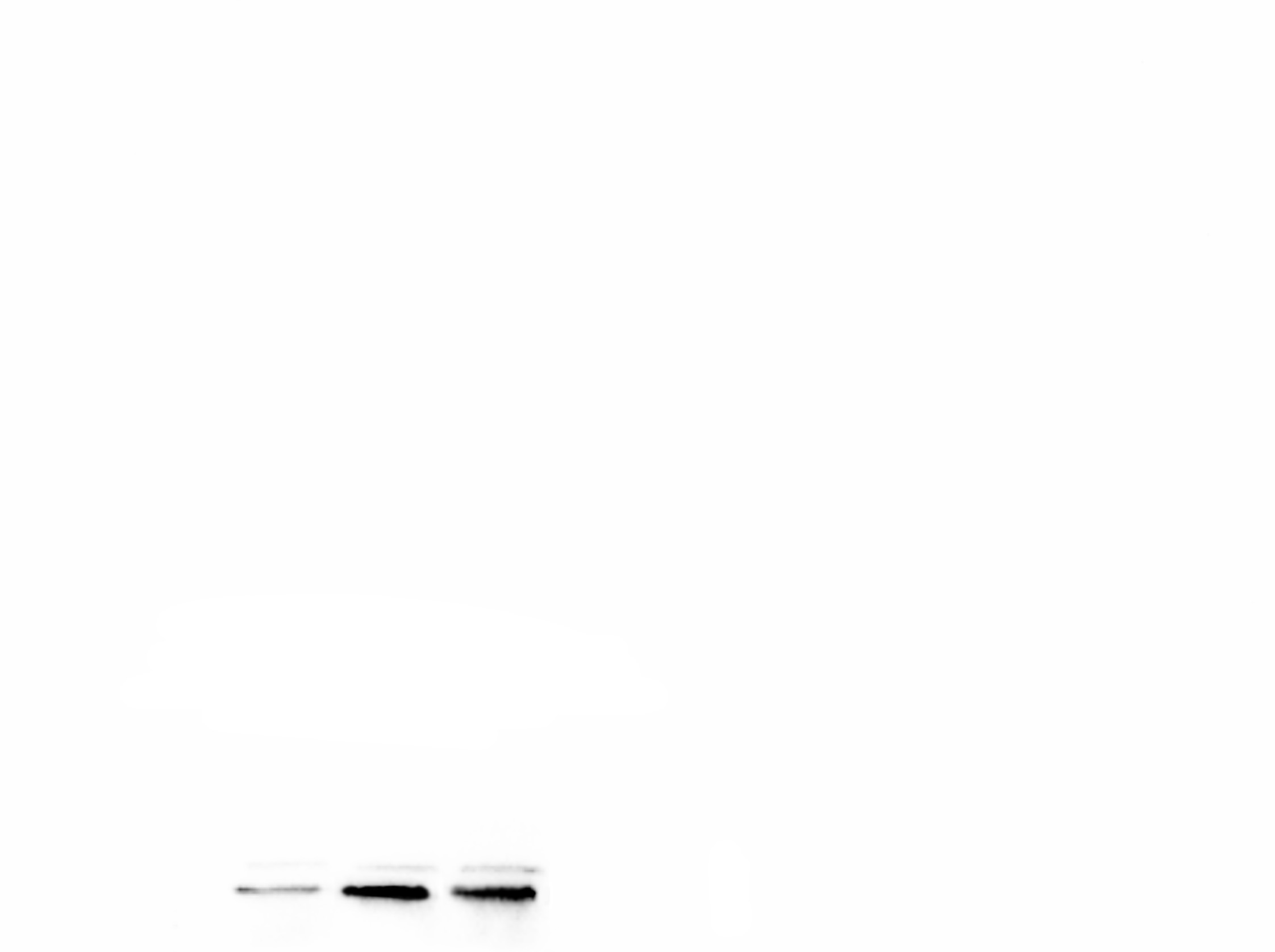

Supplement: Supplementary file 1 — Additional file 1: Fig. S1. Silencing BIRC5 inhibited cell viability, migration, invasion of PC cells under IFN-γ treatment (20 ng/mL, 24 h).PC cellswere transfected with shRNA lentiviral vectors. (A and B) Cell viability was studied by CCK-8 assay. (C and D) Wound healing assay and Transwell assay were performed to detect cell migration and invasion. The columns were presented as the mean ± SEM (n ≥ 3). * and**stood forp 0.05 and p 0.01. Fig. S2. Silencing BIRC5 depressed the aggravated tumor growth and survival of PC xenograft mice induced by IFN-γ.Penl1 cells in different groups (IFN-γ; IFN-γ + BIRC5 shRNAb) and untreated Penl1 cells (model) were subcutaneously inoculated at the right axilla (100 µL containing 1 × 10^6^ cells) after mice were anesthetized. (A) Image of tumor growth in living mice. (B, C and D) Tumor volume and tumor weight were measured and survival rate was calculated. The columns were presented as the mean ± SEM. ** stands for p 0.01 compared with model group. ## stands for p 0.01 compared with IFN-γ group. n= 5 in every group. [file 12885_2022_9500_MOESM1_ESM.zip › Supplementary/Fig.1E BIRC5.jpg]

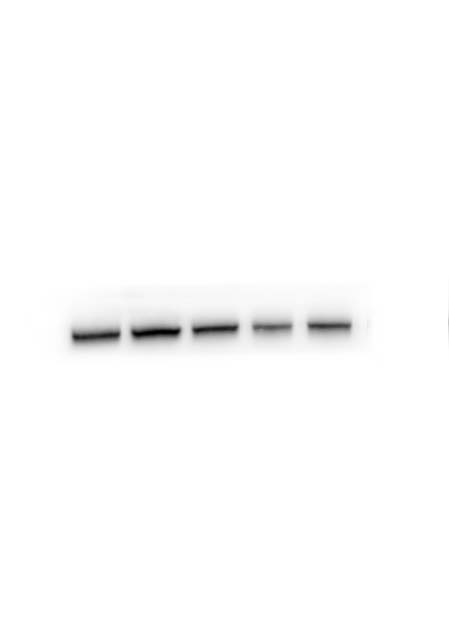

Supplement: Supplementary file 1 — Additional file 1: Fig. S1. Silencing BIRC5 inhibited cell viability, migration, invasion of PC cells under IFN-γ treatment (20 ng/mL, 24 h).PC cellswere transfected with shRNA lentiviral vectors. (A and B) Cell viability was studied by CCK-8 assay. (C and D) Wound healing assay and Transwell assay were performed to detect cell migration and invasion. The columns were presented as the mean ± SEM (n ≥ 3). * and**stood forp 0.05 and p 0.01. Fig. S2. Silencing BIRC5 depressed the aggravated tumor growth and survival of PC xenograft mice induced by IFN-γ.Penl1 cells in different groups (IFN-γ; IFN-γ + BIRC5 shRNAb) and untreated Penl1 cells (model) were subcutaneously inoculated at the right axilla (100 µL containing 1 × 10^6^ cells) after mice were anesthetized. (A) Image of tumor growth in living mice. (B, C and D) Tumor volume and tumor weight were measured and survival rate was calculated. The columns were presented as the mean ± SEM. ** stands for p 0.01 compared with model group. ## stands for p 0.01 compared with IFN-γ group. n= 5 in every group. [file 12885_2022_9500_MOESM1_ESM.zip › Supplementary/Fig.2C BIRC5.jpg]

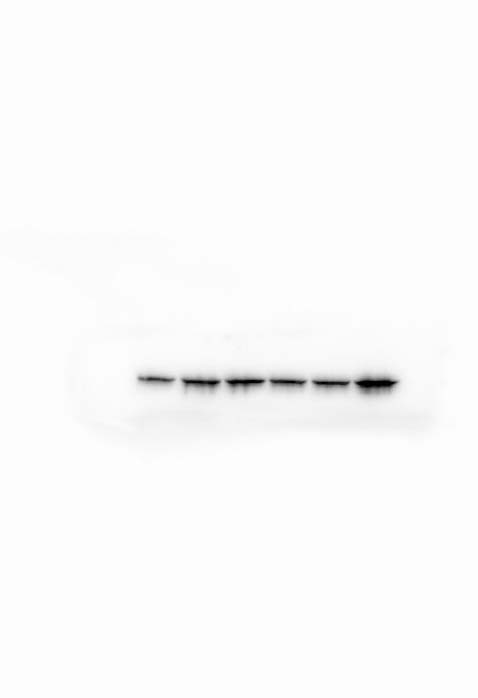

Supplement: Supplementary file 1 — Additional file 1: Fig. S1. Silencing BIRC5 inhibited cell viability, migration, invasion of PC cells under IFN-γ treatment (20 ng/mL, 24 h).PC cellswere transfected with shRNA lentiviral vectors. (A and B) Cell viability was studied by CCK-8 assay. (C and D) Wound healing assay and Transwell assay were performed to detect cell migration and invasion. The columns were presented as the mean ± SEM (n ≥ 3). * and**stood forp 0.05 and p 0.01. Fig. S2. Silencing BIRC5 depressed the aggravated tumor growth and survival of PC xenograft mice induced by IFN-γ.Penl1 cells in different groups (IFN-γ; IFN-γ + BIRC5 shRNAb) and untreated Penl1 cells (model) were subcutaneously inoculated at the right axilla (100 µL containing 1 × 10^6^ cells) after mice were anesthetized. (A) Image of tumor growth in living mice. (B, C and D) Tumor volume and tumor weight were measured and survival rate was calculated. The columns were presented as the mean ± SEM. ** stands for p 0.01 compared with model group. ## stands for p 0.01 compared with IFN-γ group. n= 5 in every group. [file 12885_2022_9500_MOESM1_ESM.zip › Supplementary/Fig.2G E-cadherin.jpg]

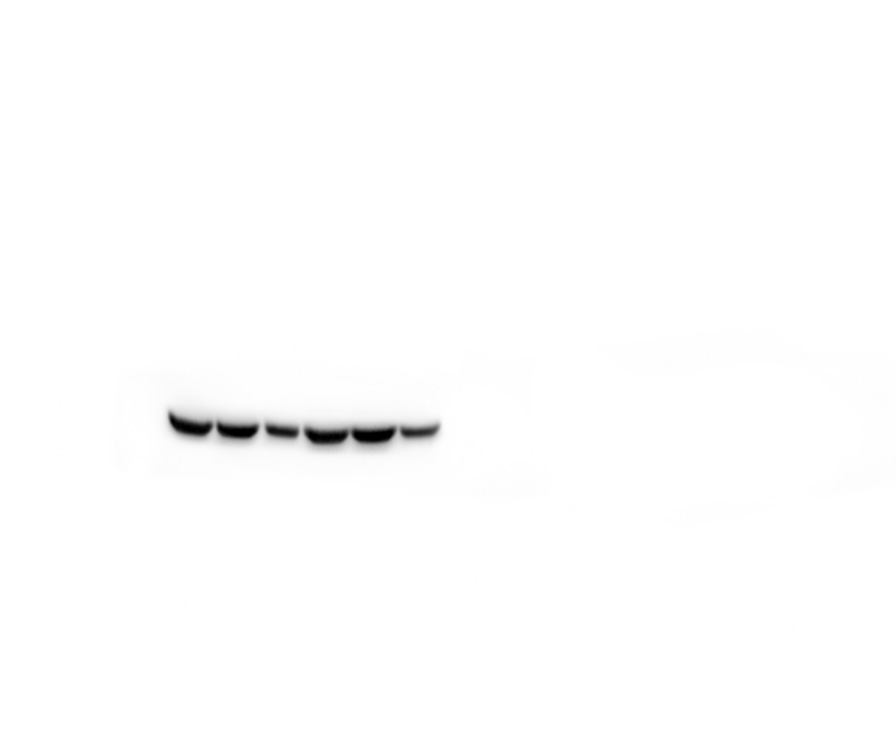

Supplement: Supplementary file 1 — Additional file 1: Fig. S1. Silencing BIRC5 inhibited cell viability, migration, invasion of PC cells under IFN-γ treatment (20 ng/mL, 24 h).PC cellswere transfected with shRNA lentiviral vectors. (A and B) Cell viability was studied by CCK-8 assay. (C and D) Wound healing assay and Transwell assay were performed to detect cell migration and invasion. The columns were presented as the mean ± SEM (n ≥ 3). * and**stood forp 0.05 and p 0.01. Fig. S2. Silencing BIRC5 depressed the aggravated tumor growth and survival of PC xenograft mice induced by IFN-γ.Penl1 cells in different groups (IFN-γ; IFN-γ + BIRC5 shRNAb) and untreated Penl1 cells (model) were subcutaneously inoculated at the right axilla (100 µL containing 1 × 10^6^ cells) after mice were anesthetized. (A) Image of tumor growth in living mice. (B, C and D) Tumor volume and tumor weight were measured and survival rate was calculated. The columns were presented as the mean ± SEM. ** stands for p 0.01 compared with model group. ## stands for p 0.01 compared with IFN-γ group. n= 5 in every group. [file 12885_2022_9500_MOESM1_ESM.zip › Supplementary/Fig.2G MMP-2.jpg]

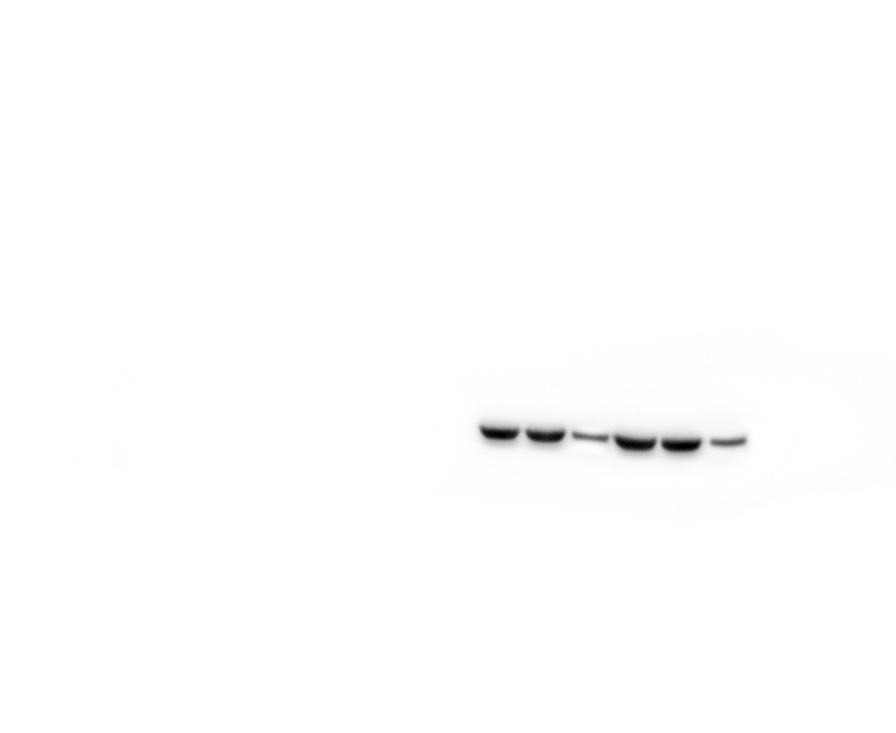

Supplement: Supplementary file 1 — Additional file 1: Fig. S1. Silencing BIRC5 inhibited cell viability, migration, invasion of PC cells under IFN-γ treatment (20 ng/mL, 24 h).PC cellswere transfected with shRNA lentiviral vectors. (A and B) Cell viability was studied by CCK-8 assay. (C and D) Wound healing assay and Transwell assay were performed to detect cell migration and invasion. The columns were presented as the mean ± SEM (n ≥ 3). * and**stood forp 0.05 and p 0.01. Fig. S2. Silencing BIRC5 depressed the aggravated tumor growth and survival of PC xenograft mice induced by IFN-γ.Penl1 cells in different groups (IFN-γ; IFN-γ + BIRC5 shRNAb) and untreated Penl1 cells (model) were subcutaneously inoculated at the right axilla (100 µL containing 1 × 10^6^ cells) after mice were anesthetized. (A) Image of tumor growth in living mice. (B, C and D) Tumor volume and tumor weight were measured and survival rate was calculated. The columns were presented as the mean ± SEM. ** stands for p 0.01 compared with model group. ## stands for p 0.01 compared with IFN-γ group. n= 5 in every group. [file 12885_2022_9500_MOESM1_ESM.zip › Supplementary/Fig.2G MMP-9.jpg]

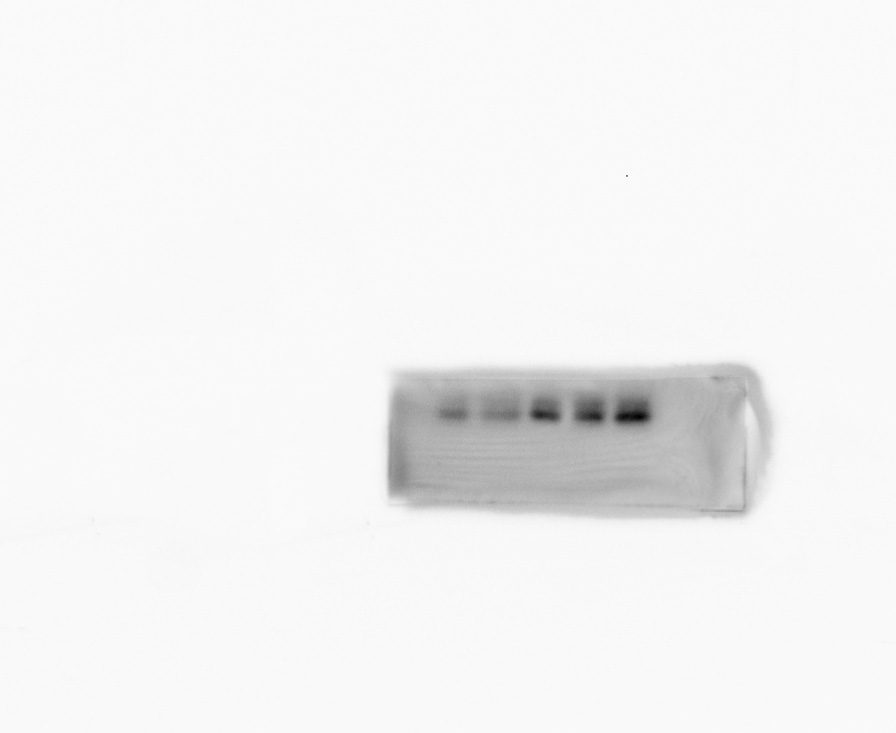

Supplement: Supplementary file 1 — Additional file 1: Fig. S1. Silencing BIRC5 inhibited cell viability, migration, invasion of PC cells under IFN-γ treatment (20 ng/mL, 24 h).PC cellswere transfected with shRNA lentiviral vectors. (A and B) Cell viability was studied by CCK-8 assay. (C and D) Wound healing assay and Transwell assay were performed to detect cell migration and invasion. The columns were presented as the mean ± SEM (n ≥ 3). * and**stood forp 0.05 and p 0.01. Fig. S2. Silencing BIRC5 depressed the aggravated tumor growth and survival of PC xenograft mice induced by IFN-γ.Penl1 cells in different groups (IFN-γ; IFN-γ + BIRC5 shRNAb) and untreated Penl1 cells (model) were subcutaneously inoculated at the right axilla (100 µL containing 1 × 10^6^ cells) after mice were anesthetized. (A) Image of tumor growth in living mice. (B, C and D) Tumor volume and tumor weight were measured and survival rate was calculated. The columns were presented as the mean ± SEM. ** stands for p 0.01 compared with model group. ## stands for p 0.01 compared with IFN-γ group. n= 5 in every group. [file 12885_2022_9500_MOESM1_ESM.zip › Supplementary/Fig.3D BIRC5.jpg]

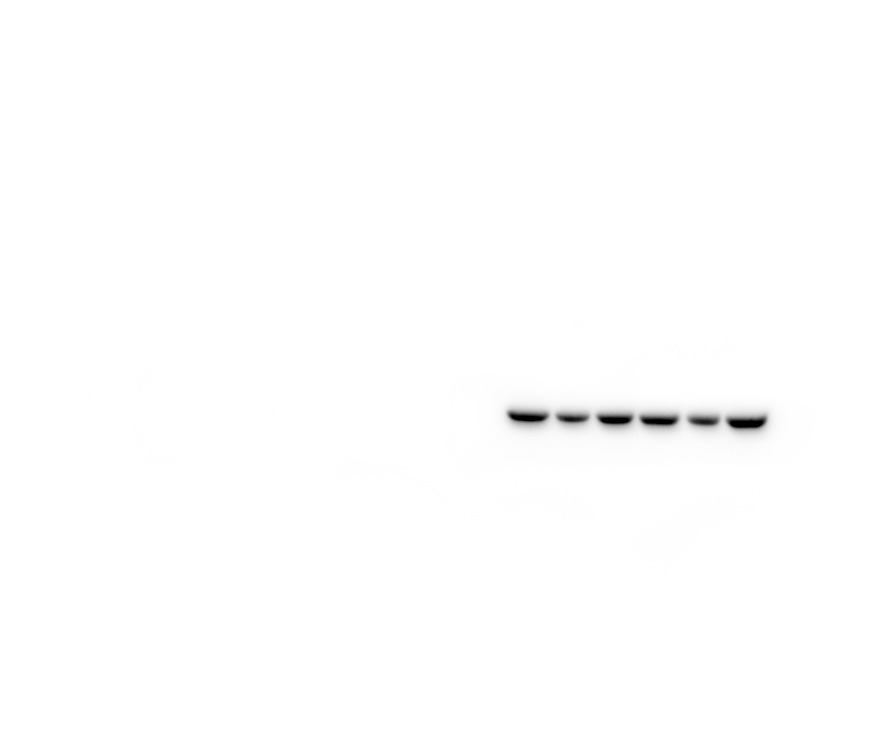

Supplement: Supplementary file 1 — Additional file 1: Fig. S1. Silencing BIRC5 inhibited cell viability, migration, invasion of PC cells under IFN-γ treatment (20 ng/mL, 24 h).PC cellswere transfected with shRNA lentiviral vectors. (A and B) Cell viability was studied by CCK-8 assay. (C and D) Wound healing assay and Transwell assay were performed to detect cell migration and invasion. The columns were presented as the mean ± SEM (n ≥ 3). * and**stood forp 0.05 and p 0.01. Fig. S2. Silencing BIRC5 depressed the aggravated tumor growth and survival of PC xenograft mice induced by IFN-γ.Penl1 cells in different groups (IFN-γ; IFN-γ + BIRC5 shRNAb) and untreated Penl1 cells (model) were subcutaneously inoculated at the right axilla (100 µL containing 1 × 10^6^ cells) after mice were anesthetized. (A) Image of tumor growth in living mice. (B, C and D) Tumor volume and tumor weight were measured and survival rate was calculated. The columns were presented as the mean ± SEM. ** stands for p 0.01 compared with model group. ## stands for p 0.01 compared with IFN-γ group. n= 5 in every group. [file 12885_2022_9500_MOESM1_ESM.zip › Supplementary/Fig.3I E-cadherin.jpg]

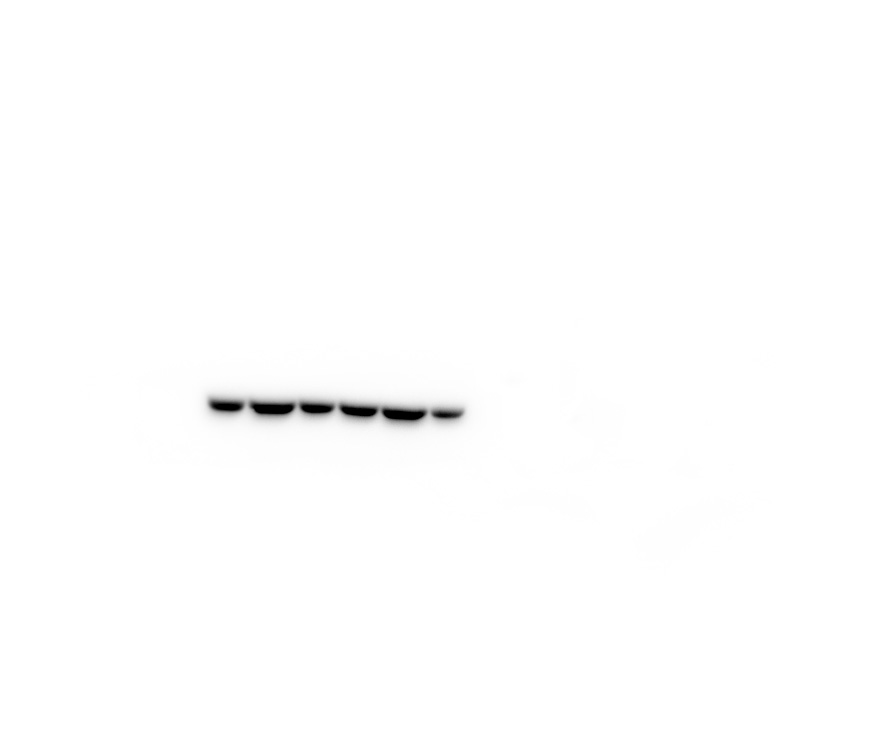

Supplement: Supplementary file 1 — Additional file 1: Fig. S1. Silencing BIRC5 inhibited cell viability, migration, invasion of PC cells under IFN-γ treatment (20 ng/mL, 24 h).PC cellswere transfected with shRNA lentiviral vectors. (A and B) Cell viability was studied by CCK-8 assay. (C and D) Wound healing assay and Transwell assay were performed to detect cell migration and invasion. The columns were presented as the mean ± SEM (n ≥ 3). * and**stood forp 0.05 and p 0.01. Fig. S2. Silencing BIRC5 depressed the aggravated tumor growth and survival of PC xenograft mice induced by IFN-γ.Penl1 cells in different groups (IFN-γ; IFN-γ + BIRC5 shRNAb) and untreated Penl1 cells (model) were subcutaneously inoculated at the right axilla (100 µL containing 1 × 10^6^ cells) after mice were anesthetized. (A) Image of tumor growth in living mice. (B, C and D) Tumor volume and tumor weight were measured and survival rate was calculated. The columns were presented as the mean ± SEM. ** stands for p 0.01 compared with model group. ## stands for p 0.01 compared with IFN-γ group. n= 5 in every group. [file 12885_2022_9500_MOESM1_ESM.zip › Supplementary/Fig.3I MMP-2.jpg]

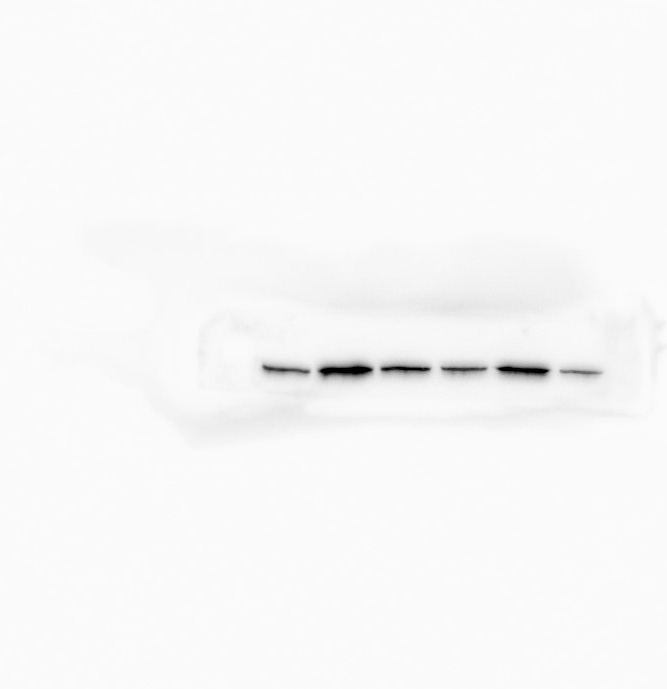

Supplement: Supplementary file 1 — Additional file 1: Fig. S1. Silencing BIRC5 inhibited cell viability, migration, invasion of PC cells under IFN-γ treatment (20 ng/mL, 24 h).PC cellswere transfected with shRNA lentiviral vectors. (A and B) Cell viability was studied by CCK-8 assay. (C and D) Wound healing assay and Transwell assay were performed to detect cell migration and invasion. The columns were presented as the mean ± SEM (n ≥ 3). * and**stood forp 0.05 and p 0.01. Fig. S2. Silencing BIRC5 depressed the aggravated tumor growth and survival of PC xenograft mice induced by IFN-γ.Penl1 cells in different groups (IFN-γ; IFN-γ + BIRC5 shRNAb) and untreated Penl1 cells (model) were subcutaneously inoculated at the right axilla (100 µL containing 1 × 10^6^ cells) after mice were anesthetized. (A) Image of tumor growth in living mice. (B, C and D) Tumor volume and tumor weight were measured and survival rate was calculated. The columns were presented as the mean ± SEM. ** stands for p 0.01 compared with model group. ## stands for p 0.01 compared with IFN-γ group. n= 5 in every group. [file 12885_2022_9500_MOESM1_ESM.zip › Supplementary/Fig.3I MMP-9.jpg]

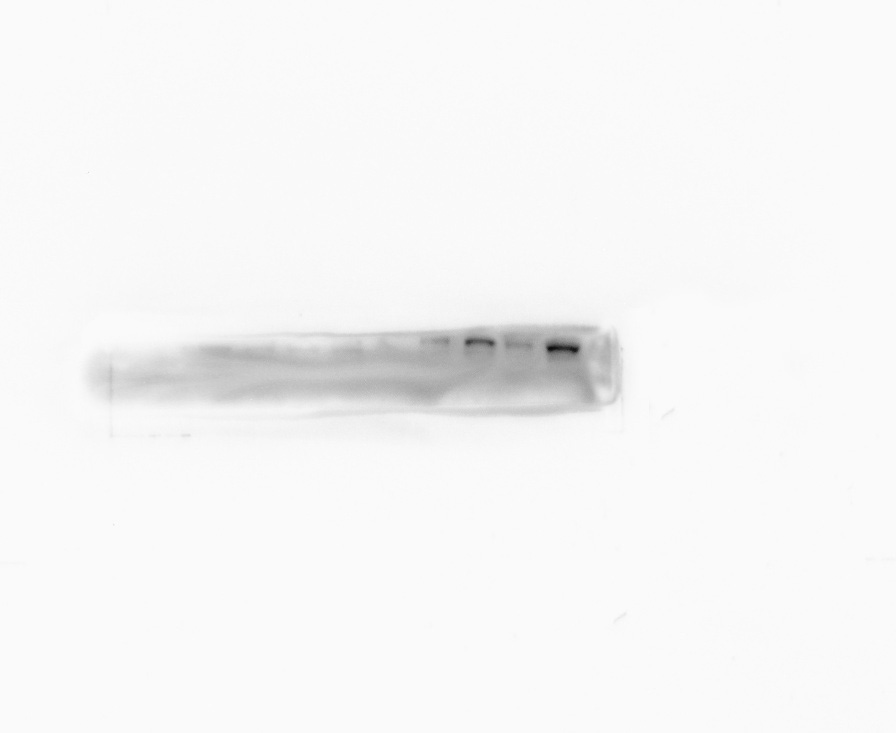

Supplement: Supplementary file 1 — Additional file 1: Fig. S1. Silencing BIRC5 inhibited cell viability, migration, invasion of PC cells under IFN-γ treatment (20 ng/mL, 24 h).PC cellswere transfected with shRNA lentiviral vectors. (A and B) Cell viability was studied by CCK-8 assay. (C and D) Wound healing assay and Transwell assay were performed to detect cell migration and invasion. The columns were presented as the mean ± SEM (n ≥ 3). * and**stood forp 0.05 and p 0.01. Fig. S2. Silencing BIRC5 depressed the aggravated tumor growth and survival of PC xenograft mice induced by IFN-γ.Penl1 cells in different groups (IFN-γ; IFN-γ + BIRC5 shRNAb) and untreated Penl1 cells (model) were subcutaneously inoculated at the right axilla (100 µL containing 1 × 10^6^ cells) after mice were anesthetized. (A) Image of tumor growth in living mice. (B, C and D) Tumor volume and tumor weight were measured and survival rate was calculated. The columns were presented as the mean ± SEM. ** stands for p 0.01 compared with model group. ## stands for p 0.01 compared with IFN-γ group. n= 5 in every group. [file 12885_2022_9500_MOESM1_ESM.zip › Supplementary/Fig.S1E E-cadherin.jpg]

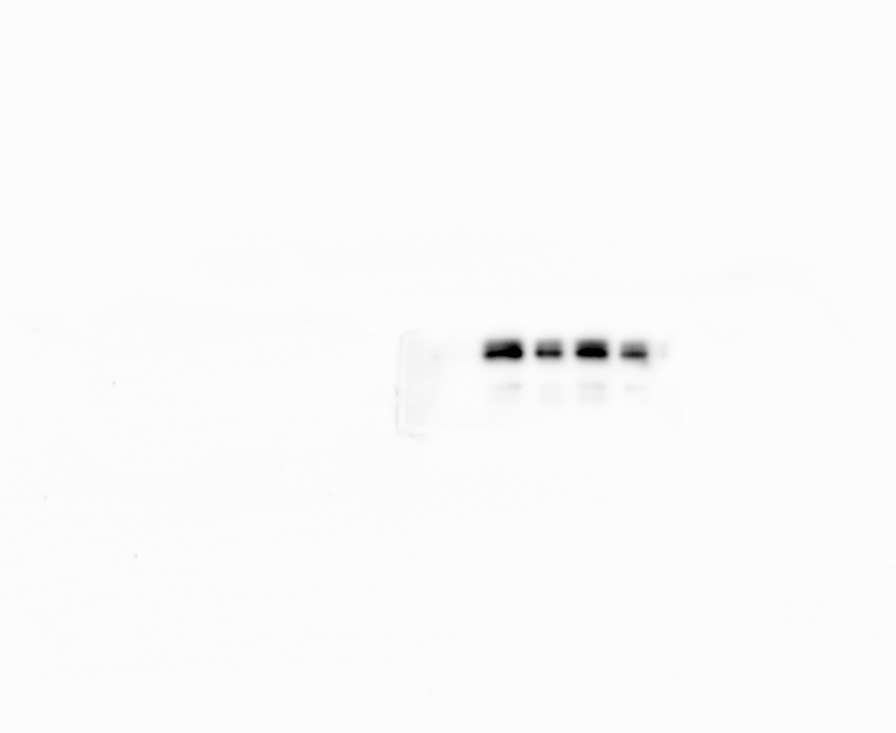

Supplement: Supplementary file 1 — Additional file 1: Fig. S1. Silencing BIRC5 inhibited cell viability, migration, invasion of PC cells under IFN-γ treatment (20 ng/mL, 24 h).PC cellswere transfected with shRNA lentiviral vectors. (A and B) Cell viability was studied by CCK-8 assay. (C and D) Wound healing assay and Transwell assay were performed to detect cell migration and invasion. The columns were presented as the mean ± SEM (n ≥ 3). * and**stood forp 0.05 and p 0.01. Fig. S2. Silencing BIRC5 depressed the aggravated tumor growth and survival of PC xenograft mice induced by IFN-γ.Penl1 cells in different groups (IFN-γ; IFN-γ + BIRC5 shRNAb) and untreated Penl1 cells (model) were subcutaneously inoculated at the right axilla (100 µL containing 1 × 10^6^ cells) after mice were anesthetized. (A) Image of tumor growth in living mice. (B, C and D) Tumor volume and tumor weight were measured and survival rate was calculated. The columns were presented as the mean ± SEM. ** stands for p 0.01 compared with model group. ## stands for p 0.01 compared with IFN-γ group. n= 5 in every group. [file 12885_2022_9500_MOESM1_ESM.zip › Supplementary/Fig.S1E MMP-2.jpg]

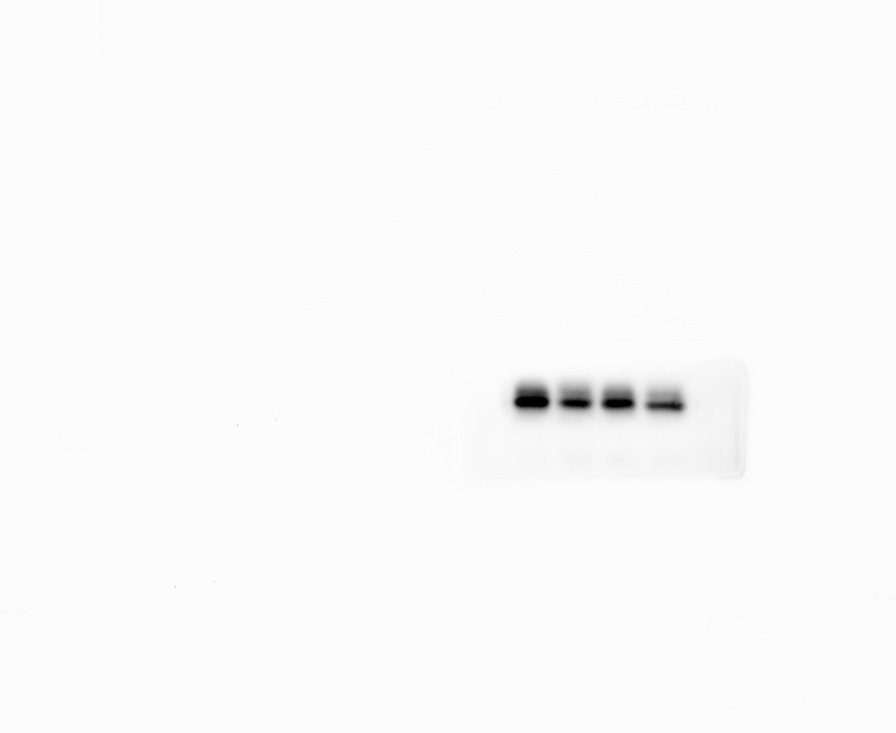

Supplement: Supplementary file 1 — Additional file 1: Fig. S1. Silencing BIRC5 inhibited cell viability, migration, invasion of PC cells under IFN-γ treatment (20 ng/mL, 24 h).PC cellswere transfected with shRNA lentiviral vectors. (A and B) Cell viability was studied by CCK-8 assay. (C and D) Wound healing assay and Transwell assay were performed to detect cell migration and invasion. The columns were presented as the mean ± SEM (n ≥ 3). * and**stood forp 0.05 and p 0.01. Fig. S2. Silencing BIRC5 depressed the aggravated tumor growth and survival of PC xenograft mice induced by IFN-γ.Penl1 cells in different groups (IFN-γ; IFN-γ + BIRC5 shRNAb) and untreated Penl1 cells (model) were subcutaneously inoculated at the right axilla (100 µL containing 1 × 10^6^ cells) after mice were anesthetized. (A) Image of tumor growth in living mice. (B, C and D) Tumor volume and tumor weight were measured and survival rate was calculated. The columns were presented as the mean ± SEM. ** stands for p 0.01 compared with model group. ## stands for p 0.01 compared with IFN-γ group. n= 5 in every group. [file 12885_2022_9500_MOESM1_ESM.zip › Supplementary/Fig.S1E MMP-9.jpg]

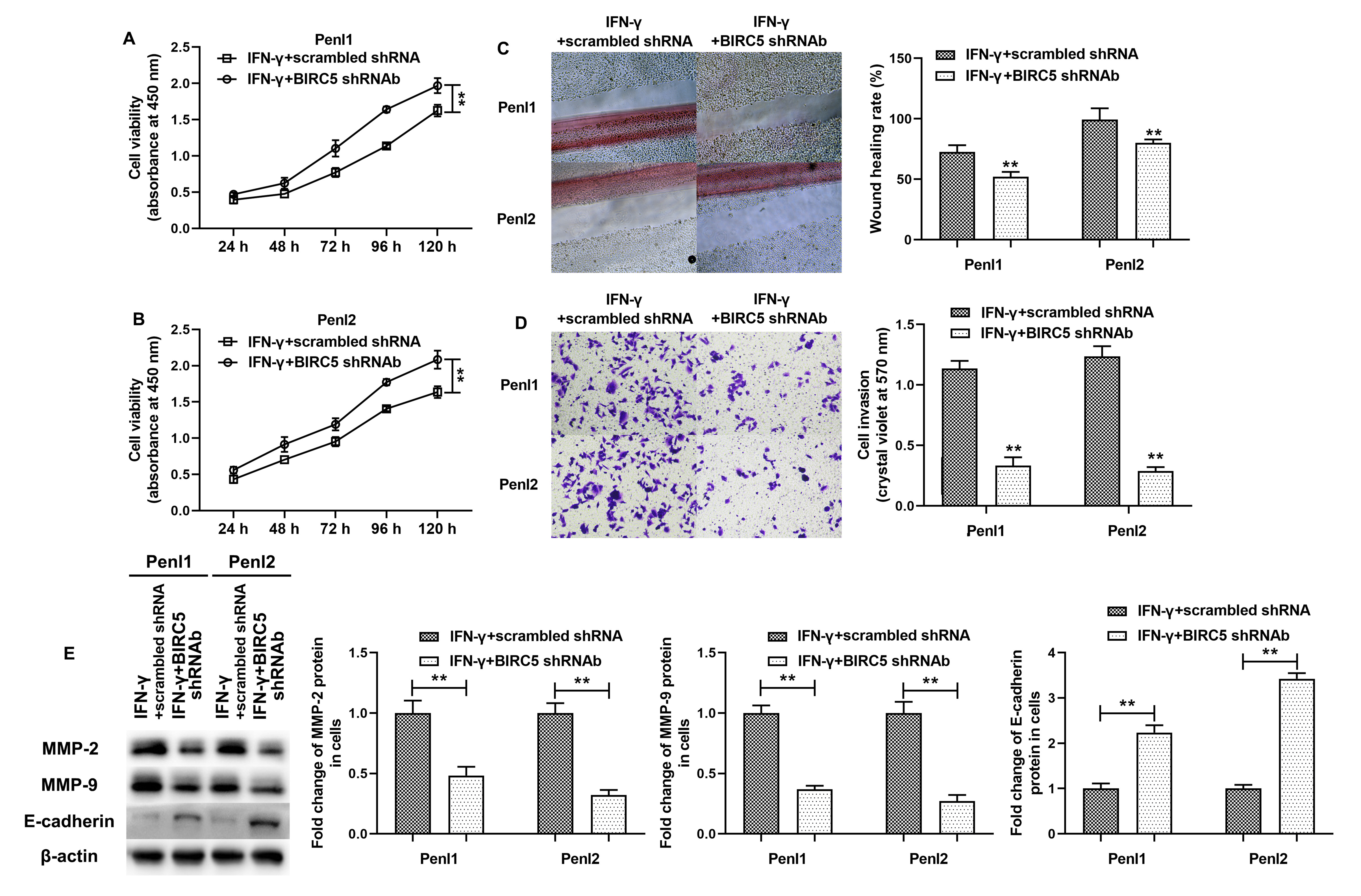

Supplement: Supplementary file 1 — Additional file 1: Fig. S1. Silencing BIRC5 inhibited cell viability, migration, invasion of PC cells under IFN-γ treatment (20 ng/mL, 24 h).PC cellswere transfected with shRNA lentiviral vectors. (A and B) Cell viability was studied by CCK-8 assay. (C and D) Wound healing assay and Transwell assay were performed to detect cell migration and invasion. The columns were presented as the mean ± SEM (n ≥ 3). * and**stood forp 0.05 and p 0.01. Fig. S2. Silencing BIRC5 depressed the aggravated tumor growth and survival of PC xenograft mice induced by IFN-γ.Penl1 cells in different groups (IFN-γ; IFN-γ + BIRC5 shRNAb) and untreated Penl1 cells (model) were subcutaneously inoculated at the right axilla (100 µL containing 1 × 10^6^ cells) after mice were anesthetized. (A) Image of tumor growth in living mice. (B, C and D) Tumor volume and tumor weight were measured and survival rate was calculated. The columns were presented as the mean ± SEM. ** stands for p 0.01 compared with model group. ## stands for p 0.01 compared with IFN-γ group. n= 5 in every group. [file 12885_2022_9500_MOESM1_ESM.zip › Supplementary/Figure S1.jpg]

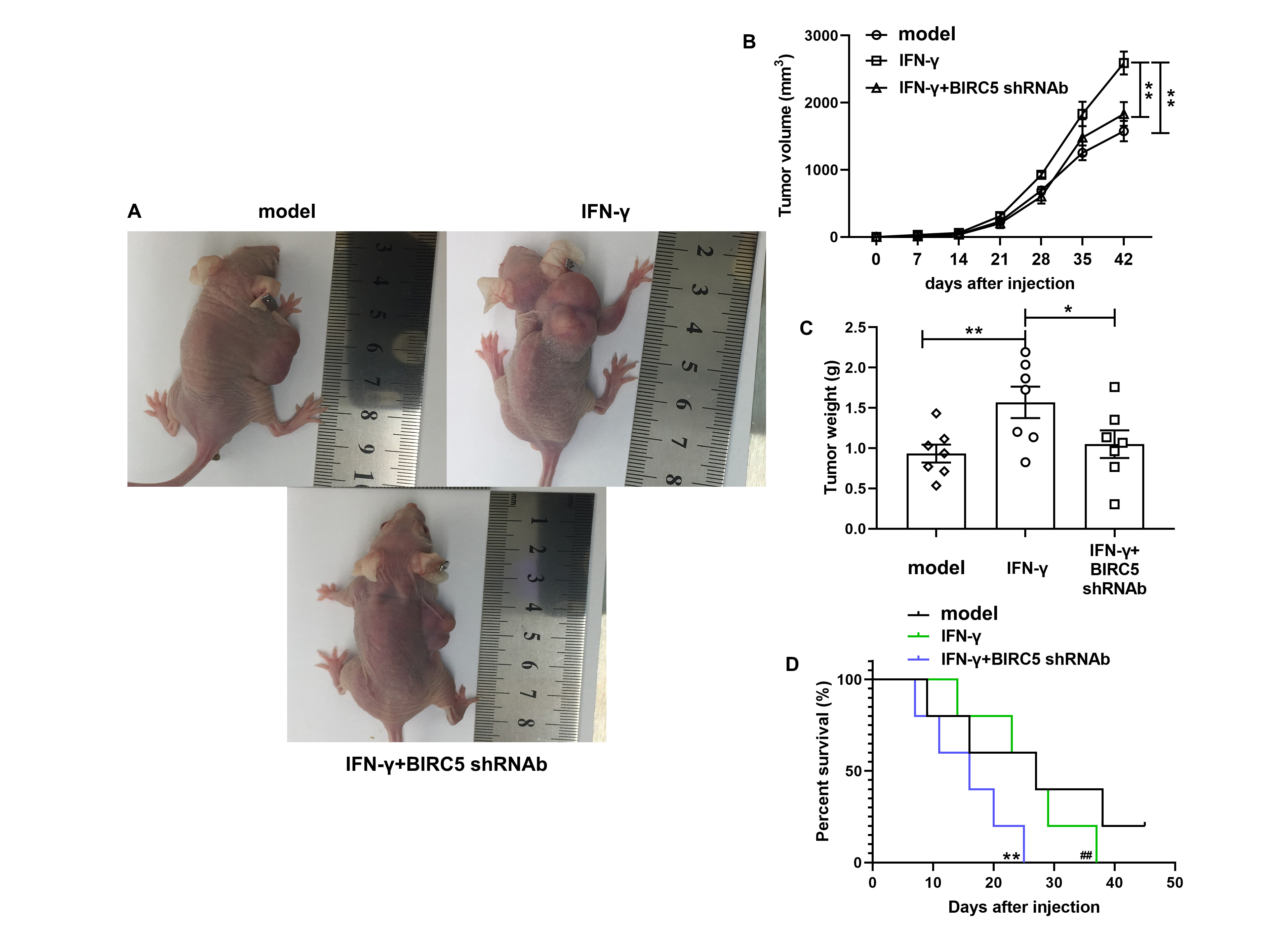

Supplement: Supplementary file 1 — Additional file 1: Fig. S1. Silencing BIRC5 inhibited cell viability, migration, invasion of PC cells under IFN-γ treatment (20 ng/mL, 24 h).PC cellswere transfected with shRNA lentiviral vectors. (A and B) Cell viability was studied by CCK-8 assay. (C and D) Wound healing assay and Transwell assay were performed to detect cell migration and invasion. The columns were presented as the mean ± SEM (n ≥ 3). * and**stood forp 0.05 and p 0.01. Fig. S2. Silencing BIRC5 depressed the aggravated tumor growth and survival of PC xenograft mice induced by IFN-γ.Penl1 cells in different groups (IFN-γ; IFN-γ + BIRC5 shRNAb) and untreated Penl1 cells (model) were subcutaneously inoculated at the right axilla (100 µL containing 1 × 10^6^ cells) after mice were anesthetized. (A) Image of tumor growth in living mice. (B, C and D) Tumor volume and tumor weight were measured and survival rate was calculated. The columns were presented as the mean ± SEM. ** stands for p 0.01 compared with model group. ## stands for p 0.01 compared with IFN-γ group. n= 5 in every group. [file 12885_2022_9500_MOESM1_ESM.zip › Supplementary/Figure S2.jpg]
